# Constructing a human complex type N-linked glycosylation pathway in *Kluyveromyces marxianus*

**DOI:** 10.1371/journal.pone.0233492

**Published:** 2020-05-29

**Authors:** Ming-Hsuan Lee, Tsui-Ling Hsu, Jinn-Jy Lin, Yu-Ju Lin, Yi-Ying Kao, Jui-Jen Chang, Wen-Hsiung Li

**Affiliations:** 1 Doctoral Degree Program in Marine Biotechnology, National Sun Yat-sen University, Kaohsiung, Taiwan; 2 Doctoral Degree Program in Marine Biotechnology, Academia Sinica, Nankang, Taipei, Taiwan; 3 Biodiversity Research Center, Academia Sinica, Nankang, Taipei, Taiwan; 4 Genomics Research Center, Academia Sinica, Nankang, Taipei, Taiwan; 5 Department of Medical Research, China Medical University Hospital, China Medical University, Taichung, Taiwan; 6 Department of Ecology and Evolution, University of Chicago, Chicago, Illinois, United States of America; National Health Research Institutes, TAIWAN

## Abstract

Glycosylation can affect various protein properties such as stability, biological activity, and immunogenicity. To produce human therapeutic proteins, a host that can produce glycoproteins with correct glycan structures is required. Microbial expression systems offer economical, rapid and serum-free production and are more amenable to genetic manipulation. In this study, we developed a protocol for CRISPR/Cas9 multiple gene knockouts and knockins in *Kluyveromyces marxianus*, a probiotic yeast with a rapid growth rate. As hyper-mannosylation is a common problem in yeast, we first knocked out the α-1,3-mannosyltransferase (*ALG3*) and α-1,6-mannosyltransferase (*OCH1*) genes to reduce mannosylation. We also knocked out the subunit of the telomeric Ku domain (*KU70*) to increase the homologous recombination efficiency of *K*. *marxianus*. In addition, we knocked in the *MdsI* (α-1,2-mannosidase) gene to reduce mannosylation and the *GnTI* (β-1,2-*N*-acetylglucosaminyltransferase I) and *GnTII* genes to produce human N-glycan structures. We finally obtained two strains that can produce low amounts of the core N-glycan Man_3_GlcNAc_2_ and the human complex N-glycan Man_3_GlcNAc_4_, where Man is mannose and GlcNAc is N-acetylglucosamine. This study lays a cornerstone of glycosylation engineering in *K*. *marxianus* toward producing human glycoproteins.

## Introduction

Proper protein glycosylation is important because glycosylation affects the stability, biological activity, and immunogenicity of a protein [1]. Many clinically approved therapeutic proteins are glycosylated. Therefore, efforts to engineer glycosylation pathways have been made in a wide variety of cell types including bacterial, fungal, and mammalian cells [2, 3]. Mammalian cell lines are usually preferred because they produce complex glycans similar to those in humans. However, the requirements for complex nutrients in culture media and the special growth conditions impede the application of mammalian cell lines in glycan engineering [4]. In contrast, microbial expression systems have advantages, such as growth in serum-free media and simpler genetic engineering procedures [5].

The purpose of this study is to construct a N-linked glycosylation pathway in *Kluyveromyces marxianus* to produce the human complex type N-glycan Man_3_GlcNAc_4_ (i.e., GlcNAc_2_Man_3_GlcNAc_2_, where Glc is glucose, Man is mannose, and GlcNAc is N-acetylglucosamine), which is a precursor to more complex human glycan structures (S1 Fig). To produce Man_3_GlcNAc_4_, we design an engineering strategy after comparing the human and yeast glycosylation pathways (right side of S1 Fig). This is also one of the three humanization pathways suggested by Kim *et al*. [6]. We propose first to delete the *ALG3* (α-1,3- mannosyltransferase) gene because it is responsible for the first mannosylation step in the endoplasmic reticulum (ER) and its deletion will prevent the conversion of Man_5_GlcNAc_2_ to Man_6_GlcNAc_2_ (S1 Fig). Also, we plan to delete the *OCH1* (α-1,6-mannosyltransferase) gene at the same time because this step will prevent the addition of mannose to the branched outer chain of Man_5_GlcNAc_2_. Then, the insertion of a *MdsI* (α-1,2-mannosidase) gene into *K*. *marxianus* will delete two α-1,2-mannoses from Man_5_GlcNAc_2_, leading to the glycan core Man_3_GlcNAc_2_. Finally, the insertion of *GnTI* (β-1,2-*N*-acetylglucosaminyltransferase I) and *GnTII* genes will produce Man_3_GlcNAc_4_, the glycan structure we want to produce. To achieve this purpose, we have developed a CRISPR/Cas9 system in *K*. *marxianus*, which we call “PCK” (Protocol for CRISPR/Cas9 multiple gene knockouts and knockins) (S2 Fig) (see Methods).

Much effort has been made to use yeasts to produce human glycoproteins [7]. In fact, a complete humanization pathway has been established in *P*. *postoris* [8, 9]. However, the production rate is very low and the glycan heterogeneity is high. Thus, much further effort is needed. Also, in many previous studies the pathway was constructed on plasmids, such as GlycoSwitch [10], while in this study the pathway genes will be integrated into the genome. Moreover, while most previous studies mainly relied on traditional transformation techniques for gene knockouts and knockins, we utilize the CRISPR/Cas9 technique.

In this study, we choose *K*. *marxianus* as the host for engineering glycosylation pathways because it is a probiotic yeast that has safety certifications (QPS and EFSA) [11] and it has a rapid growth rate, can use both hexose and pentose sugars, and is a toxin- and heat-tolerance [12–14]. In our previous study, we have obtained a haploid Cas9-carrying strain, which is named “*K*. *marxianus* α2” [15]. This strain will be the starting point of our study.

## Results

### Analysis of N-glycans in different yeasts

**Fig 1:**
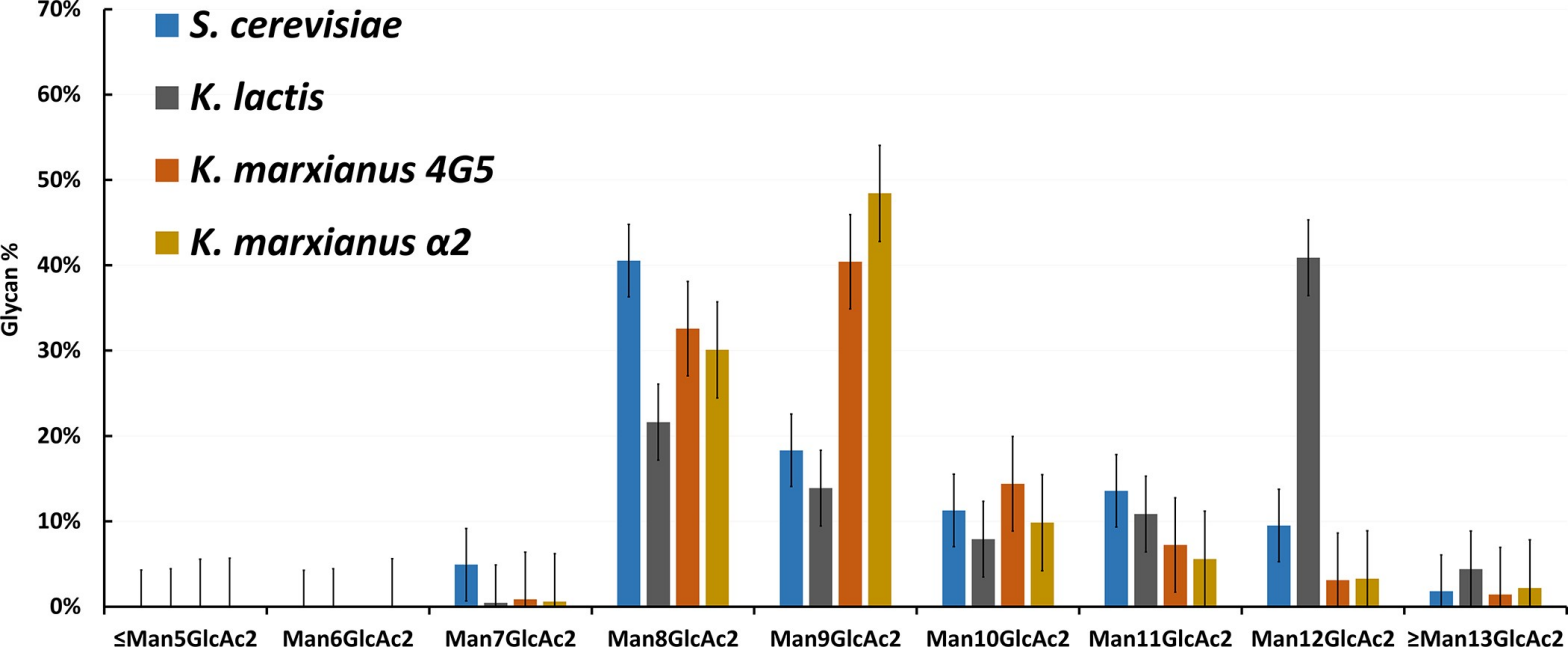
Analysis of N-glycans in *S*. *cerevisiae*, *K*. *lactis*, and *K*. *marxianus* 4G5 and *K*. *marxianus* α2.

Many yeasts, such as *Pichia pastoris* and *Saccharomyces cerevisiae*, produce hyper-mannosylated proteins [16]. We analyzed the N-glycans in *S*. *cerevisiae*, *K*. *lactis*, *K*. *marxianus* 4G5 (a wild-type diploid), and *K*. *marxianus* α2, a Cas9-carrying haploid strain derived from 4G5 [15] (Fig 1). The glycan profile differs significantly among these yeasts, but in each strain most of the glycans contained 7–12 mannoses and two N-acetylhexosamines (i.e., Man_7-12_GlcNAc_2_). In *K*. *marxianus* 4G5, the N-glycans with <10 mannoses account for 74% and those with ≧10 glycans accounted for 26% of the glycans. The same was true for *K*. *marxianus* α2. Although the proportions of Man_7_GlcNAc_2_ and Man_8_GlcNAc_2_ were higher in *S*. *cerevisiae* than in *K*. *marxianus* 4G5, the proportion of Man_8_GlcNAc_2_ was much lower in S. cerevisiae. Thus, in S. cerevisiae, the N-glycans with <10 mannoses accounted for only 64% while those with ≧10 glycans accounted for 36% of the total N-glycans. K. lactis belongs to the same genus as *K*. *marxianus*, but only 36% of its N-glycans had <10 mannoses, while 64% had ≧10 mannoses. Thus, *K*. *marxianus* has weaker mannosylation than *S*. *cerevisiae* and *K*. *lacti*s.

### Generation of the Man_5_GlcNAc_2_ N-glycan

Our first step was to produce the Man_5_GlcNAc_2_ N-glycan core in *K*. *marxianus* α2 (S1 Fig); it is the glycan flipped from the cytosolic face to the luminal face of the ER [17]. For this purpose, we planned to knock out *ALG3* and *OCH1* in *K*. *marxianus* α2 (S1 Fig). Also, to increase the frequency of the DNA integration via homologous recombination (HR) [18], we planned to knock out *KU70*, which is involved in the non-homologous end joining (NHEJ) pathway. At the same time, we also wanted to knockin *GnTII* (~3.5 kb) and a donor DNA fragment HR-Blank (4.5 kb) for testing the knockin of a relatively long DNA fragment. Thus, we simultaneously knocked out *ALG*3, *OCH1* and *KU70* and knocked in *GnTII* and HR-Blank into the gRNA cutting sites on the *KU70* and *ALG3* genes, respectively, using PCK and the G418 selection marker gene. For the above knockouts and knockins, we designed the gRNAs to target the conserved regions of the *KU70*, *ALG3* and *OCH1* genes using the CRISPOR software [19] and constructed them on T&A vectors (S2 and S3A Figs, S1 Table). We named the strain obtained “*K*. *marxianus* αO3-I2”, which has the genotype *ku70*::*GnTII*, *alg3*::HR-Blank, and *och1*::(+33bp) (S2 Fig). (We use “O” and “I” to denote “knockout” and “knockin”, respectively, so “O3” means “3 knockouts” and “I2” means “2 knockins”.)

We confirmed the insertion of 33 bp in the *OCH1* gene by PCR and sequencing (S4 Fig) and the knockins of *GnTII* and HR-Blank by PCR (S5 Fig). We also determined that this strain was an α haploid type by mating-type confirmation (S5G Fig).

**Fig 2:**
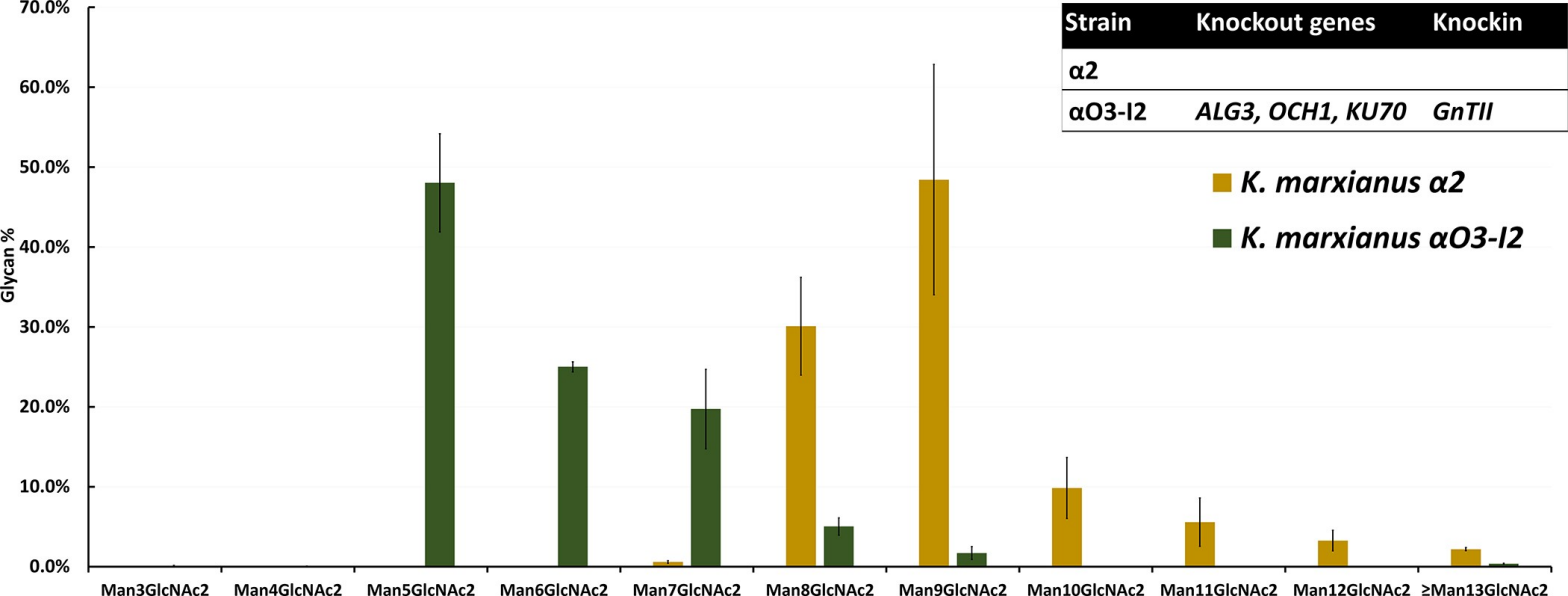
Profiles of N-glycans in *K*. *marxianus* α2 and αO3-I2 strains. The proportion of Man_5_GlcNAc_2_ in *K*. *marxianus* αO3-I2 (the left dark green color bar) was greatly increased compared to that in *K*. *marxianus* α2. The number of replicates in each experiment is 3 (N = 3).

We conducted LC-MS analyses of glycan profiles and found that in *K*. *marxianus* αO3-I2, the proportion of Man_5_GlcNAc_2_ increased from 0% to 48±6%, compared to *K*. *marxianus* α2 (Fig 2). Also, the proportions of Man_6_GlcNAc_2_ and Man_7_GlcNAc_2_ increased significantly while the proportions of glycan forms with > 7 mannoses were greatly reduced (Fig 2). These observations can be taken as the effect of the deletion of *ALG3* and *OCH1* on mannosylation in *K*. *marxianus* αO3-I2.

### Generation of Man_3_GlcNAc_2_ and Man_3_GlcNAc_4_

As *Trichoderma reesei* α-1,2-mannosidase (MdsI) effectively removes α-1,2-mannose residues on the Man_5_GlcNAc_2_ structure in the Golgi [20], we used PCK to simultaneously knock out *URA3* and knock in the *MdsI* gene into *K*. *marxianus* αO3-I2 with donor DNA fragment HR-*ura3*-*MdsI*, where HR-*ura3* stands for homologous recombination site in the disrupted *URA3* gene. We knocked out the *URA3* gene to provide a 5-FOA (5-Fluoroorotic acid) selection. The resultant *K*. *marxianus* αO4-I3 strain was obtained through auxotrophy after a PCR check (S5 Fig). In another construct, we simultaneously knocked in two donor DNA fragments HR-*ura3*-*MdsI* and HR-*ura3*-*GnTI* at the *URA3* gRNA cleavage sites; the two fragments recombined into a fragment of ~8 kb. We call the new strain “*K*. *marxianus* αO4-I4”, which carries both the *GnT I* and *II* genes for adding GlcNAc residues to Man_3_GlcNAc_2_. We confirmed the insertions of these genes into the *URA3* gene by PCR (S5 Fig).

**Fig 3:**
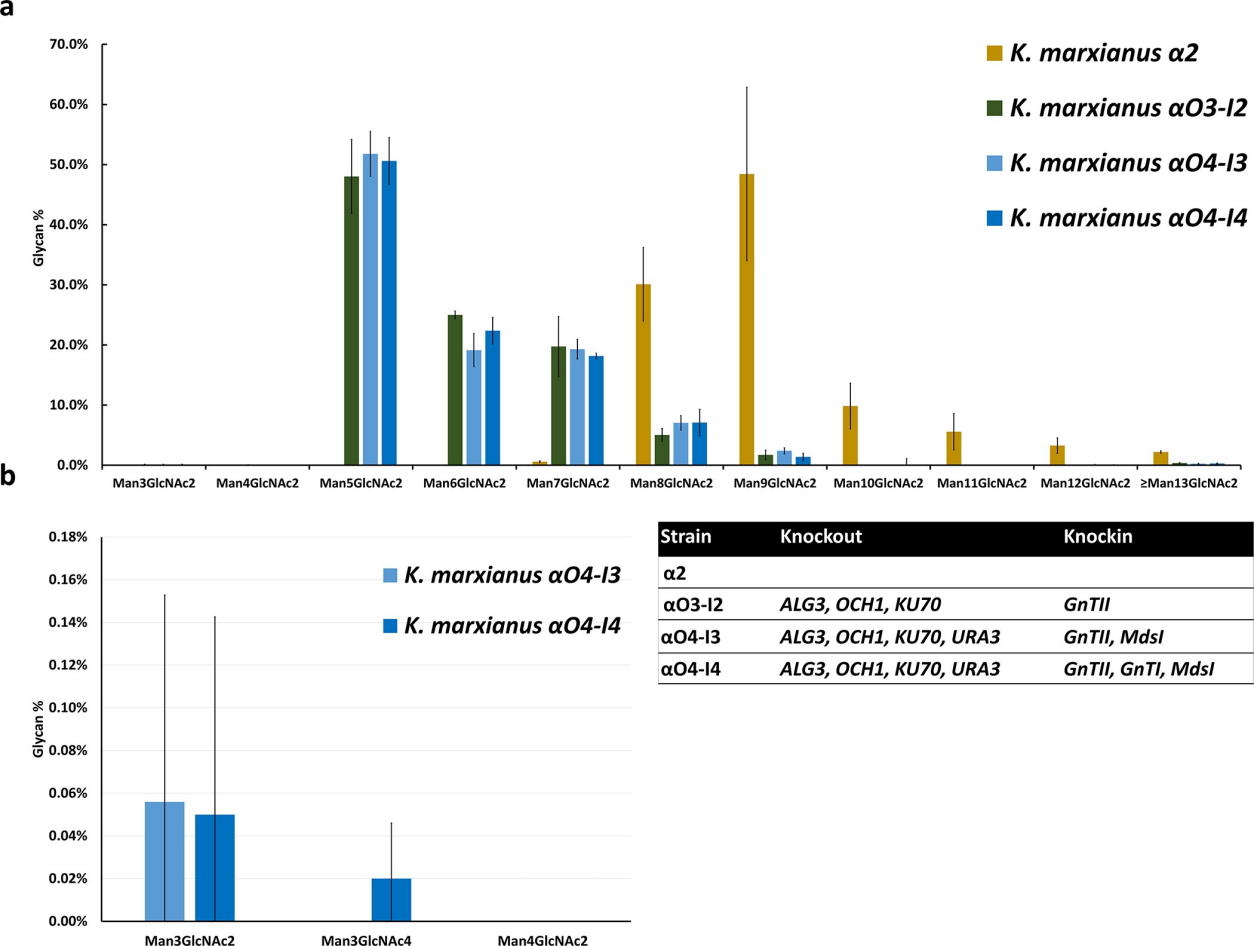
Comparison of the N-glycan profiles in four engineered strains. (a) The N-glycan profiles in the *K*. *marxianus* α2, αO3-I2, αO4-I3, and αO4-I4 strains. Much higher proportions of Man_5_GlcNAc_2_, Man_6_GlcNAc_2_ and Man_7_GlcNAc_2_ are observed in the αO3-I2, αO4-I3 and αO4-I4 strains than the α2 strain. (b) Proportions of Man_3_GlcNAc_2_ and Man_3_GlcNAc_4_ glycans in the αO4-I3 and αO4-I4 strains.

Our glycan profile analyses revealed much higher proportions of Man_5_GlcNAc_2_, Man_6_GlcNAc_2_ and Man_7_GlcNAc_2_ in *K*. *marxianus* αO3-I3, αO4-I3 and αO4-I4 than in *K*. *marxianus* α2; that is, as expected, the number of mannoses per glycan has been greatly reduced. Moreover, the glycan profile of *K*. *marxianus* αO4-I3 showed a very low amount (0.06±0.09%) of Man_3_GlcNAc_2_ (Fig 3B), and a ~3.74% increase in Man_5_GlcNAc_2_ compared to *K*. *marxianus* αO3-I2 (Fig 3A). *K*. *marxianus* αO4-I4 showed very small amounts (0.05±0.09% and 0.02±0.03%) of Man_3_GlcNAc_2_ and Man_3_GlcNAc_4_ (Fig 3B), which is the desired glycan structure.

### Increasing the accumulation of Man_3_GlcNAc_2_

**Fig 4:**
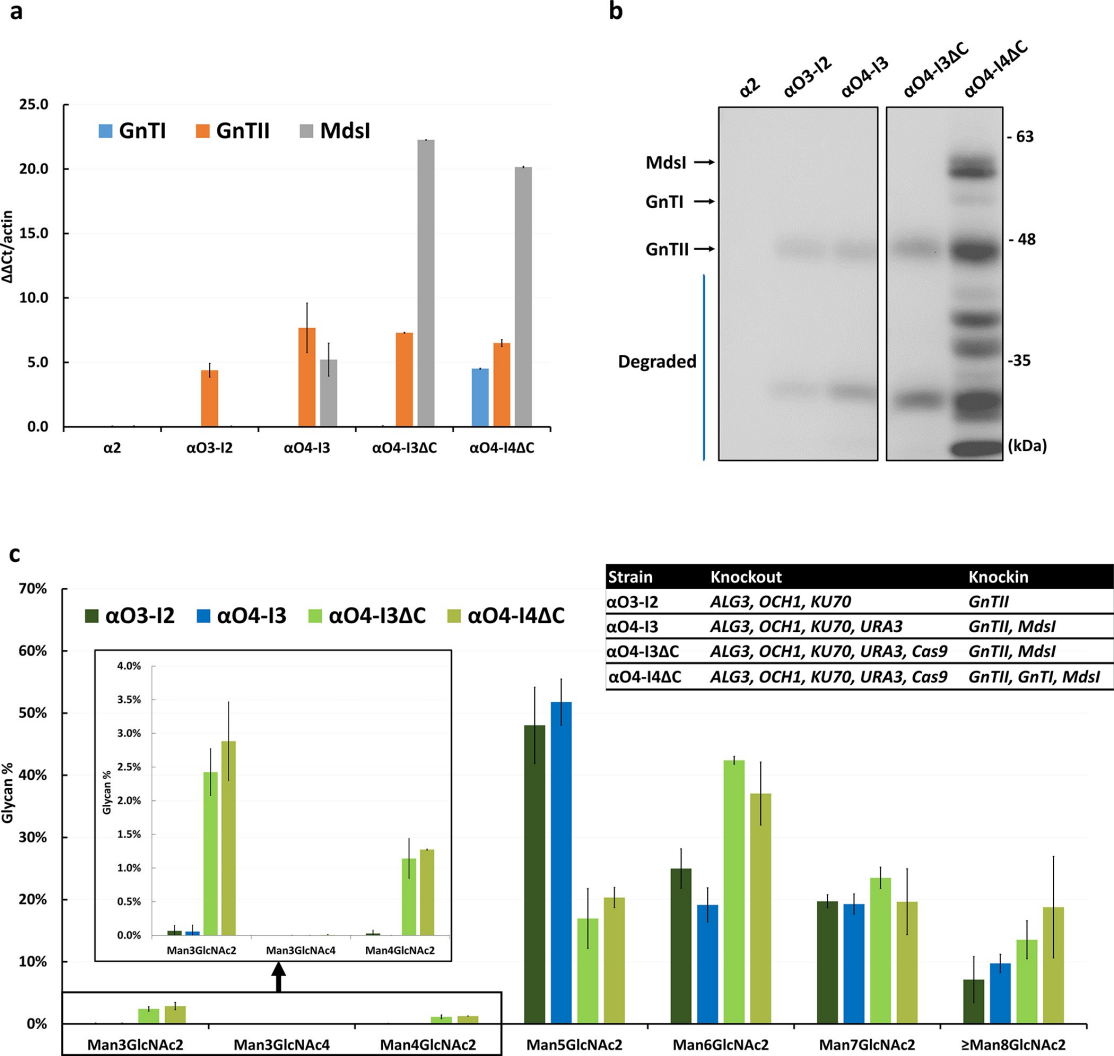
Analyses of RNA expression, protein expression level and N-glycan profile in *K*. *marxianus* α2, αO3-I2, αO4-I3, αO4-I3ΔC and αO4-I4ΔC. (a) The RNA expression levels of the *GnTI*, *GnTII*, and *MdsI* genes. The qRT-PCR used the endogenous actin gene as the reference. (b) Western blot analysis of protein expression of the *MdsI*, *GnTI* and *GnTII* genes. The molecular weights of GnTI, GnTII, and MdsI are 51, 45 and 62 kDa, respectively. The arrows indicate the MdsI, GnTI, and GnTII proteins. The blue line indicates degraded proteins. (c) The glycan profiles in four engineered strains. The zoom-in figure shows the profiles of Man_3_GlcNAc_2_, Man_3_GlcNAc_4_, and Man_4_GlcNAc_2_ in the four engineered strains. The number of experimental replicates was 2 for αO4-I3ΔC and αO4-I4ΔC and 3 for the other strains.

**Fig 5:**
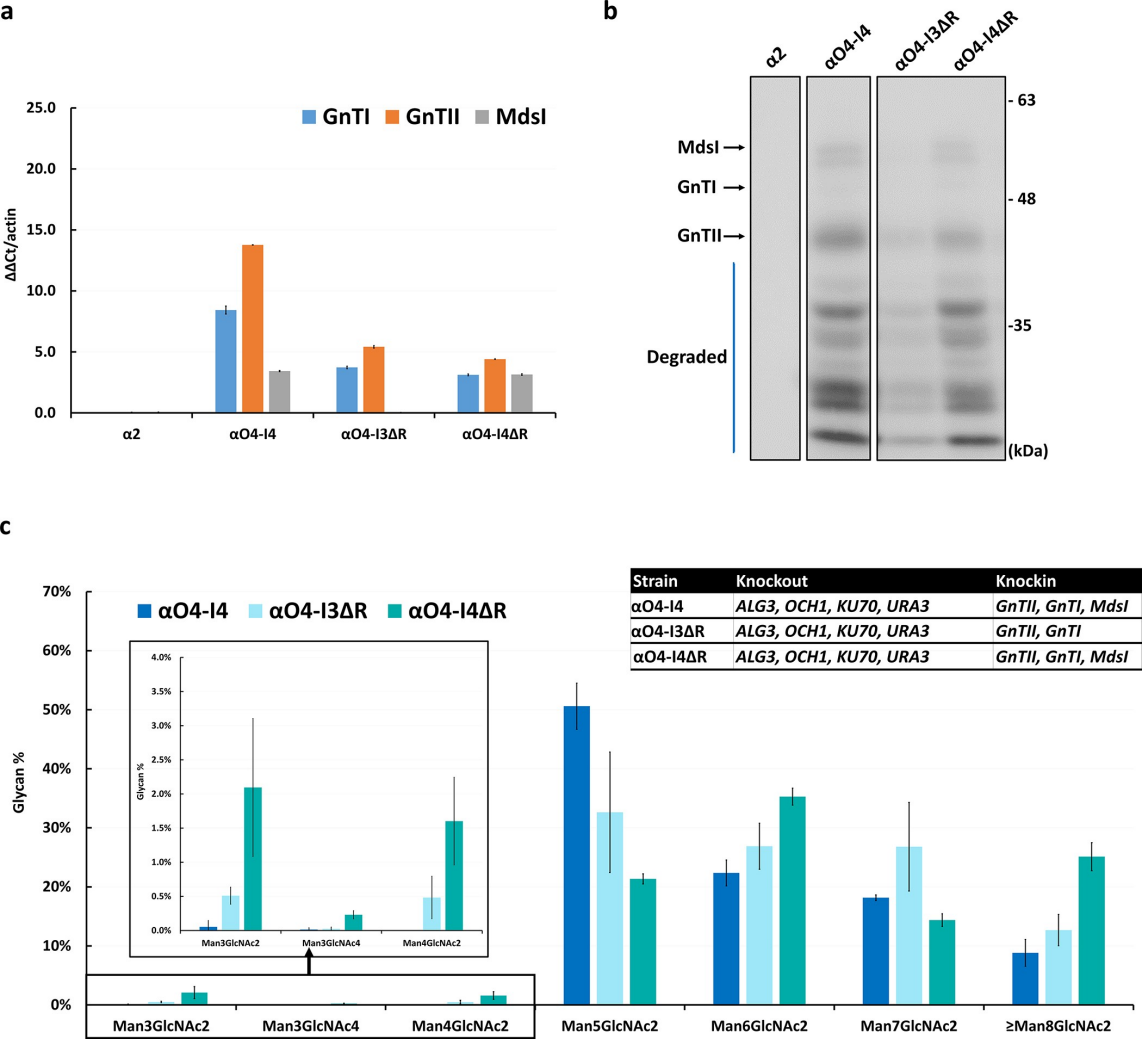
Analyses of RNA expression level, protein expression level and N-glycan profile in *K*. *marxianus* αO4-I4, αO4-I3ΔR and αO4-I4ΔR. (a) The RNA expression levels of *GnTI*, *GnTII*, and *MdsI*. The qRT-PCR used the endogenous actin gene as the reference. (b) Western blot analysis of protein expression of *MdsI*, *GnTI* and *GnTII*. The arrows indicate the MdsI, GnTI, and GnTII proteins. The blue line indicates degraded proteins. (c) The glycan profiles in the four engineered strains. The zoom-in figure shows the profiles of Man_3_GlcNAc_2_, Man_3_GlcNAc_4_, and Man_4_GlcNAc_2_ in the four strains. The number of replicates was 3 for each experiment.

We noted above that the proportions of Man_3_GlcNAc_2_ in the *K*. *marxianus* αO4-I3 and αO4-I4 strains, both of which include the *MdsI* gene, were extremely low. This could be due to a low expression level of the *MdsI* gene in these two strains. Our RNA analysis showed that the expression level of *MdsI* was lower than that of *GnTII* in *K*. *marxianus* αO4-I3 and αO4-I4 (Figs 4A and 5A), so it might not be high enough for producing the amount of Mds1 required for cleaving α1,2 mannose. This could be in part because the *Cas9*, *MdsI*, *GnTI* and *GnTII* genes all used the *LAC4* promoter (P_LAC4_). To test this possibility, we constructed two new strains: (1) *K*. *marxianus* αO4-I3ΔC, which was derived from *K*. *marxianus* αO4-I3 by knocking out the multiple *Cas9* genes and the *zeocin*, *hygromycin* and *G418* resistance genes, using no selection marker but by cell dilution, and (2) *K*. *marxianus* αO4-I4ΔC, which was derived from *K*. *marxianus* αO4-I3ΔC by knocking in the *GnTI* gene, using the *G418* resistance gene as the selection marker. The expression of *MdsI* was indeed greatly increased in these two new strains (Fig 4A). The production of Man_3_GlcNAc_2_ was also increased to 2.43±0.25% in αO4-I3ΔC and 2.88±0.6% in αO4-I4ΔC (Fig 4C). These proportions were still very low, likely because of severe protein degradation (Fig 4B) (see discussion). The *GnTI* gene was expressed at a fairly high level in *K*. *marxianus* αO4-I4ΔC (Fig 4A), but only 0.01±0.008% Man_3_GlcNAc_4_ was detected (Fig 4C). This might be because only a faint band was seen in the western blot analysis of protein expression, probably because of protein degradation (Fig 4B).

In another effort, we deleted the hygromycin and *G418* resistance genes in *K*. *marxianus* αO4-I4. Unfortunately, the *MdsI* gene was lost in the new strain; we call this strain without *MdsI* “*K*. *marxianus* αO4-I3ΔR”. We knocked the *MdsI* gene into *K*. *marxianus* αO4-I3ΔR, using the *G418* resistance gene as the selection marker, and obtained the new strain *K*. *marxianus* αO4-I4ΔR. The *MdsI* gene was inserted in the *LAC4* promoter region in αO4-I4ΔR, while inside the *URA3* gene in αO4-I4. The expression level of *MdsI* was almost the same in αO4-I4 and αO4-I4ΔR (Fig 5A) and the MdsI protein was seen as a faint band in both strains (Fig 5B). However, while αO4-I4 produced only 0.05±0.09% Man_3_GlcNAc_2_ and 0.02±0.03% Man_3_GlcNAc_4_ (Fig 3), αO4-I4ΔR produced 2.10±1.24% Man_3_GlcNAc_2_ and 0.23±0.07% Man_3_GlcNAc_4_ (Fig 5C). That is, αO4-I4ΔR showed increased production of both Man_3_GlcNAc_2_ and Man_3_GlcNAc_4_, albeit at low levels.

## Discussion

In this study, we developed a CRISPR/Cas9 system, called PCK, for gene knockouts and knockins in *K*. *marxianus*. Our protocol uses linearized DNA fragments to facilitate transformation by electroporation. We showed that PCK could be used to simultaneously knock out three genes and knock in two genes. Moreover, our DNA cassette design enables two or more DNA fragments to recombine into one fragment after they are transformed into the cell. Thus, PCK is a useful tool for genome editing.

We found that *K*. *marxianus* 4G5 has weaker hypermannosylation than *S*. *cerevisiae* and *K*. *lactis* (Fig 1). *K*. *marxianus* α2 and *K*. *marxianus* 4G5 have similar glycan profiles (Fig 1) and growth rates [15]. Moreover, *K*. *marxianus* α2 is a haploid and carries multiple *Cas9* genes, which can facilitate genetic manipulations. Thus, it is a suitable host for our purpose.

According to our glycosylation engineering plan (S1 Fig), we first knocked out the *ALG3* and *OCH1* genes in *K*. *marxianus* α2 (and at the same time knocked in the *GnTII* gene) to reduce hypermannosylation and the resultant strain *K*. *marxianus* αO3-I2 indeed showed a great reduction in the average number of mannoses per glycan (Fig 2). However, more than 50% of the glycans in *K*. *marxianus* αO3-I2 still carried more than 5 mannoses, suggesting that other enzymes can add mannoses to glycans.

To convert Man_5_GlcNAc_2_ to Man_3_GlcNAc_2_, the core glycan, we knocked the *MdsI* gene into *K*. *marxianus* αO3-I2 and obtained the *K*. *marxianus* αO4-I3 strain, which could produce Man_3_GlcNAc_2_, albeit at a very low level (only 0.06±0.09%) (Fig 3). To produce the human complex glycan Man_3_GlcNAc_4_, our target glycan structure, we knocked the *GnTI* and *MdsI* genes into *K*. *marxianus* αO3-I2, which already carried the *GnTII* gene, and obtained the *K*. *marxianus* αO4-I4 strain. This strain indeed could produce Man_3_GlcNAc_4_, although at a very low level (only 0.02±0.03%).

To raise the productions of Man_3_GlcNAc_2_ and Man_3_GlcNAc_4_, we constructed three new strains. First, we derived *K*. *marxianus* αO4-I3ΔC from *K*. *marxianus* αO4-I3 by knocking out the multiple *Cas9* genes and the antibiotics zeocin, hygromycin and G418 resistance genes. As the *Cas9* genes and *MdsI* and *GnTII* genes in αO4-I3 were all driven by the P_LAC4_, knocking out the *Cas9* genes and their promoters greatly increased the expression level of *MdsI*, although not that of *GnTII*, leading to an increase in the production of Man_3_GlcNAc_2_ from ~0% to 2.43±0.25%. Second, we knocked in the *GnTI* gene into αO4-I3ΔC to obtain αO4-I4ΔC, which could produce 2.88±0.58% Man_3_GlcNAc_2_ and 0.01±0.008% Man_3_GlcNAc_4_, which is our target glycan structure. Third, we derived αO4-I4ΔR from αO4-I4. Although the only difference between the two strains is that αO4-I4 contains the *hygromycin* resistance gene while αO4-I4ΔR does not, αO4-I4 could produce only 0.05±0.09% Man_3_GlcNAc_2_ and 0.02±0.03% Man_3_GlcNAc_4_ (Fig 3B) but αO4-I4ΔR could produce 2.10±1.24% Man_3_GlcNAc_2_ and 0.23±0.07% Man_3_GlcNAc_4_ (Fig 5C).

Our study is still substantially behind studies in other yeasts. For example, the proportions of Man_3_GlcNAc_2_ and Man_3_GlcNAc_4_ are 2.10% and 0.23%, respectively, in our study but were 1.92% and 35.48% in *S*. *cerevisia*e [21]. Thus, much effort remains to be made.

As proof of concept, our data do indicate that the glycosylation engineering steps we proposed (S1 Fig) can indeed lead to the production of the human complex glycan Man_3_GlcNAc_4_, although at a very low level. Thus, our challenge now is how to raise the production of Man_3_GlcNAc_4_. Our Western blot analysis of the MdsI, GntI and GnTII proteins in *K*. *marxianus* αO4-I4ΔC and *K*. *marxianus* αO4-I4ΔR suggested that severe degradation of these proteins was likely a reason for the low production of Man_3_GlcNAc_4_. Therefore, our next task is to reduce protein degradation.

Protein degradation is usually due to peptide cleavage by proteases and disruption of protease genes has been found to increase the yield of recombinant peptides expressed in yeasts [22–24]. Of particular relevance is the eukaryotic secretory aspartyl protease family (pfam00026) that includes cathepsin D, pepsin, renin, penicillopepsin, and fungal yapsins (Yps’s). For example, disrupting the *Yps1* gene in *S*. *cerevisiae* increased the yield of heterologous peptides. From the *K*. *marxianus* genome, we have identified five proteins homologous to pfam00026 aspartyl proteases (i.e., Yps1p, Yps7p, Pep4p, Prb1p and Bar1p). *K*. *marxianus* Yps1p (KLMA_20534) and Yps7p (KLMA_40262) are yapsin family proteases that are putatively attached to the plasma membrane or cell wall via a glycosylphosphatidylinositol anchor. Bar1p (KLMA_50468) is homologous to a *S*. *cerevisiae* periplasmic protease that mediates pheromone degradation and cleaves and inactivates α-factor [23]. Pep4p (KLMA_70025) is a soluble vacuolar protease (proteinase A) required for the post-translational precursor maturation of vacuolar proteinases that are important for protein turnover after oxidative damage [25]. *K*. *marxianus* Prb1p (KLMA_80029) is a yeast vacuolar protease (proteinase B) and its role is similar to Pep4p [26]. Destruction of these proteases could effectively increase the peptide yield [27]. We shall first knock out the genes for *Yps1* [28] and/or *Pep4* [27, 29] to see if the productions of Man_3_GlcNAc_2_ and Man_3_GlcNAc_4_ are increased. If this is still not sufficient to explain the low productions, we will consider the other two proteases or search for other proteases.

Another possible reason for the low production of Man_3_GlcNAc_2_ in glycoengineered yeasts is the phosphorylation of glycans, which adds phosphates to α1,2-linked mannose residues at four sites of N-glycans, preventing the hydrolysis of terminal α1,2-linked mannose by MdsI [10, 30]. In our data, the proportions of phosphorylated glycans were much higher in *K*. *marxianus* glycoengineered strains than *K*. *marxianus* α2 and phosphorylation occurred mainly on Man_≥5_GlcNAc_2_ (S8 Fig). It has been shown that N-glycan mannosylphosphorylation can be abolished in *S*. *cerevisiae*, *P*. *pastoris*, and *Y*. *lipolytica* by the disruption of the *MNN4* and/or *MNN14* genes [31–33]. Our bioinformatics analysis revealed that *K*. *marxianus* lost the *MNN6* gene and that *K*. *marxianus MNN4* (KLMA 30052) and *MMN14* (KLMA_10282, PNO1) are homologous *S*. *cerevisiae MNN4* and *MMN14*, respectively. We therefore plan to knock out these two genes in our glycoengineered strains (e.g., *K*. *marxianus* αO4-I4ΔR) to see if it can prevent or reduce phosphorylation of glycans.

## Materials and methods

### Prediction of gRNAs

The gRNAs to target the *KU70*, *OCH1*, *ALG3*, *URA3* and *S*. *cerevisiae ADHI* promoter (P_ADHI_) and terminator were predicted as in Lee *et al*. [15]. We constructed gRNA vectors of pMH-g1~g12 using PCR and ligation (S1 and S2 Tables).

### Yeast strains, media and culture conditions

The *Kluyveromyces marxianu*s α2 strain (*MATα*, Δ*MATα3*) used in this study was created from the *K*. *marxianu*s 4G5 diploid strain [15]. It is a haploid Cas9-carrying strain. The culture conditions used in this study were as described previously [15, 34]. The genotypes of all strains used in this study are shown in S2 Table. For the selection of gene knockout strains, the YPG medium with 200 μg/mL of G418 was used, if G418 was used as the selection marker, and the YPG medium with 0.1% 5-FOA (5-Fluoroorotic acid, Watson Biotechnology Co.) was used for selection of *URA3* knockout strains.

The knockout mutants were streaked out for 5 generations for colony purification and then cultured in YPGU (YPG with 0.1% uracil) or YPGUC (YPGU with 0.2% CaCl_2_·H2O) media at 30°C for 36 hours for growth test and glycan analysis.

### The PCK protocol

The PCK (protocol for CRISPR/Cas9 multiple gene knockouts and knockins) protocol starts with the *K*. *marxianus* α2 strain as the host. The protocol consists of four steps (S2 Fig): (1) RNA design and construction. We use CRISPOR (http://crispor.tefor.net/) to exclude off-targets and improve on-target efficiency, and the RNAfold Webserver (http://rna.tbi.univie.ac.at//cgi-bin/RNAWebSuite/RNAfold.cgi) to predict gRNA secondary structure. We select 1~3 gRNAs for each target gene. We construct each gRNA on the T&A vector. The double-stranded gRNA cassette is amplified by PCR using the M13 primer pairs. (2) Gene or donor DNA cassette design and construction. A homologous recombination sequence of ~60 bp is designed at the left end and another sequence at the right end of each cassette. We use the P_LAC4_ to drive the *GnTI*, *GnTII*, and *MdsI* genes. Each selection marker gene is driven by the P_ADHI_ derived from *S*. *cerevisiae*. The primer pairs of the recombination fragments are ligated to the head and tail of the target gRNA or gene cassette for PCR amplification. (3) Transformation of gRNA and gene cassettes. The *Cas9* gene expression is continued for 6 to 12 hours. Linearized gRNA, donor DNA fragments, and a selection marker gene are simultaneously transformed into yeast cells by electroporation. (4) Colony selection. A colony screening can be done using antibiotics or nutrition gene selection.

### Plasmid construction

The plasmids used in this study are listed in S1 Table. The commercial vector pKLAC2 (*K*. *lactis* Protein Expression Kit [35], New England Biolabs, MA) was used as the gene expression backbone with the *G418* selection marker. We synthesized the genes by optimizing its codon usage for *K*. *marxianus* (Protech Technology Enterprise Co., Ltd.). Restriction cutting sites on the plasmid pMH1-pMH3 are marked in S7 Fig. The following plasmids were used in this study:

1. The HDEL-tagged *T*. *reesei* α-1,2-mannosidase (*MdsI*) [Genbank^®^ Accession No. AF212153] had proven effective in hydrolyzing α-1,2-linked mannose residues *in vivo* in fungus [36, 37]. The plasmid pMH-1 contained the 3’ end of the P_LAC4_, the signal peptide sequence of the *S*. *cerevisiae* α-mating factor, the open reading frame of the *T*. *reesei* α-1,2-mannosidase cloned in frame, the coding sequence for HDEL and a stop codon. The coding sequence for a 12x His-Tag was inserted between the sequences coding for the catalytic domain and the HDEL signal (S7A Fig).
2. The pMH-2 plasmid was constructed according to a previous study [38] and contained the signal peptide sequence of the *S*. *cerevisiae* P283 Mnn9p AA 1–40 [GenBank: EWH15443.1][10], the open reading frame of the *Homo sapiens* β-1,2-*N*-acetylglucosaminyltransferase (*MGAT1*, GnTIΔ43) (NCBI accession: NM_001114617.1) in frame [38–40], 12x His-Tag and a stop codon (S7B Fig).
3. The pMH-3 plasmid was constructed according to a previous study [41] and contained the signal peptide sequence of the *S*. *cerevisiae* YJM1399 Mnn2p AA 1–36 [GenBank: AJQ15701.1] [42], the open reading frame of *Rattus norvegicus* β-1,2-*N*-acetylglucosaminyltransferase (*MGAT2*, GnTIIΔ88) the [NCBI Reference Sequence: NM_053604.2] [43] in frame, 12x His-Tag and a stop codon (S7C Fig).

All oligonucleotide primers used for PCR-based assembly of DNA fragments and for checking gene insertions are listed in S3 Table. The gRNA cassettes were constructed in pMHg1-g12 plasmids with the SNR52 promoter and SUP4 terminator (S1 Table). All PCR amplification of gRNA and donor DNA cassettes was performed in 2X Green tag buffer (EmeraldAm Max HS PCR Master Mix, TaKaRa) in a total reaction volume of 30 μl. Thermo-cycling consisted of incubation at 95°C for 3 min followed by 35 cycles of successive incubations at 95°C for 10 secs, 55°C for 30 secs (5 min for donor DNA) and 68°C for 30 secs (8 min for donor DNA). After thermos-cycling, a final extension was performed at 68°C for 10 min.

### Validation of gene knockouts and knockins

If the size of a DNA fragment knockout was smaller than 50 bp, the validation was carried out by sequencing. Each target gene insertion of the HR-cassette at the gRNA cutting site was checked by PCR. After culturing, we lysed the cells in QE buffer (QuickExtract^TM^ DNA Extraction Solution, Lucigen) at 65°C for 30 min and 95°C for 15 min. The total of 2 μl DNA with the specific primer pair and Green Tag PCR Mix solution (EmeraldAm Max HS PCR Master Mix, TaKaRa) was used for PCR reaction. The PCR reaction was conducted at 95°C for 3 min followed by 35 cycles of incubation at 95°C for 10 sec, 55°C for 20 sec (6 min for long fragment) and 68°C for 1 min (8 min for long fragment). The final extension was performed at 68°C for 10 min.

### Western blot and qRT-PCR

Western blot analysis and qRT-PCR were conducted as in Lee *et al*.[15]. His-Tag antibody (HRP-conlugated 6*His, His-Tag Mouse McAb, Proteintech) was diluted 1: 5000 for western blot. The qRT-PCR primer pairs used in this study are listed in S1 Table.

### Mass spectrometry and data analysis

Yeast cell pellets were collected after overnight culturing in the volume of 50 ml and then re-suspended in 30 ml of 10 mM HEPES buffer. Lysates were prepared through the disruption process six times in a Microfluidizer^®^ processor (Microfluidics Co., Westwood, MA), followed by centrifugation at 6,000 rpm for 5 min. The supernatant was passed through a 0.45 μm filter (Pall Co., Port Washington, NY) and the protein concentration was measured by Pierce BCA assay (Thermo Fisher Scientific, San Jose, CA). Lysates were subjected to in-solution tryptic digestion with filter-assisted sample preparation (FASP) method [44] and subsequently treated with PNGase F to release N-glycans. Released glycans were cleaned up by C18 cartridges and detected by LC-ESI-MS on a LTQ Orbitrap XL ETD mass spectrometer (Thermo Fisher Scientific) equipped with Waters Acquity UPLC (Waters, Milford, MA) system, and a PGC HT column (1.0 mm x 150 mm, 3 μm, Thermo Fisher Scientific) with homemade heating oven (190°C). The gradient employed was 98% buffer A/2% buffer B at 2 min 40% buffer A/to 60% buffer B at 20 min with a flow rate of 250 μL/min, where buffer A was 0.1% formic acid/H_2_O, and buffer B was 0.1% formic acid/80% acetonitrile. Survey full-scan MS condition: mass range m/z 500–2000, resolution 15,000 at m/z 400. The most intense ions were sequentially isolated for HCD (Resolution 7500). Electrospray voltage was maintained at 4.0 kV and the capillary temperature was set at 275°C. The m/z corresponding to the N-glycan was analyzed by GlycoWorkbench [45] through the search in the Consortium of Glycomics (CFG) N-glycan database, and the relative intensity of each ion was used for the calculation to give the percentage of each glycan.

## Supporting information

S1 Fig The proposed steps to construct a N-linked glycosylation pathway to produce GlcNAc_2_Man_3_GlcNAc_2_ in *K*. *marxianus*. The glycosylation pathway in the ER is the same from yeast to human. The human glycosylation in the Golgi (left panel) requires the following glycosyltransferases [46]: GnTI (β-1,2-N-acetylglucosaminyltransferase I), GnTII (β-1,2-N-acetylglucosaminyltransferase II), GalT (β-1,4-galactosyltransferase I) and ST (sialyltransferase). In *S*. *cerevisiae* (middle panel), hypermannosylation is initiated in the Golgi by the α1,6-mannosyltransferase (OCH1), which adds mannoses onto the α1,3 branch of the tri-mannose core, generating an α1,6-linked mannose branch. Additional mannosyltransferases subsequently extend this branch, leading to hypermannosylation. In this study we propose to knock out the *ALG3* and *OCH1* genes and knock in *MdsI* (α-1,2-mannosidase), *GnTI* and *GnTII* to produce the complex glycoform GlcNAc_2_Man_3_GlcNAc_2_.
(TIF)

S2 Fig The PCK protocol. Step 1: gRNA design and construction. To exclude off-targets and improve on-target efficiency, we use the CRISPOR software (http://crispor.tefor.net/). For gRNA secondary structure calculation, we use the bioinformatical tool RNAfold Webserver(http://rna.tbi.univie.ac.at/cgi-bin/RNAWebSuite/RNAfold.cgi). The designed gRNA is constructed on the T&A vector. The double-stranded gRNA expression cassette is amplified by PCR using the M13 primer pairs. Step 2: Gene or donor DNA cassette design and construction. A homologous recombination sequence of ~60 bp is designed at the left and right ends of each gRNA site. The primer pairs of the recombination fragments are ligated to the head and tail positions of the target gene cassette for PCR amplification. Step 3: Transformation of gRNA and gene cassettes. Cas9 gene expression is continued for 6 to 12 hours. Linearized gRNA, donor DNA fragments and a selection marker are transformed into yeast cells by electroporation. Step 4: Colony selection. We select strains from the plate.
(TIF)

S3 Fig The gRNA cutting sites on the *KU*70, *OCH*1, *ALG*3, and *URA*3 genes and *S*. *cerevisiae* ADHI promoter (P_ADHI_) and terminator (used for transforming the *G418*, *zeocin*, *hygromycin B* and *Cas9* genes). The gRNA cutting sites were also the homologous recombination sites for donor DNA cassettes. (a) The gRNA cutting sites in different target genes. The arrows indicate the gRNA cutting sites. A forward strand DNA is indicated by a right arrow and a reversed strand DNA is indicated by a left arrow. (b) A donor DNA fragment was inserted into the gRNA cutting site in the target gene by homologous recombination. The gray part indicates the gRNA cutting sites of target genes that were also used for the homologous recombination (HR) for the gene expression cassettes. (c) Six gRNA sites were designed in *S*. *cerevisiae* P_ADHI_ and terminator, which were used for designing antibiotic gene cassettes. Note that the *Cas9* coding region is in front of a *zeocin* cassette and is repeated in the P_LAC4_ region. When the *zeocin* cassette is cut, the area of P_LAC4_ will be rearranged, giving rise a chance to remove the *Cas9* gene.
(TIF)

S4 Fig The *OCH1* coding sequences of the αO3-I2 strains. The blue color indicates the original sequence and the red color indicates the regions with insertion or deletion. The αO3-I2 strain contains the 33 bp insertion at the *OCH1* gRNA cutting site.
(TIF)

S5 Fig Validation of the insertions of donor DNAs in transformants by PCR. N: negative control; M: DNA marker. Lane 1: the 4G5 wild type, Lanes 2–4: strains not used in this paper; Lane 5: Cas9-carrying *K*. *marxianus* α2; Lane 6: *K*. *marxianus* αO3-I2, Lane 7: *K*. *marxianus* αO4-I3, Lane 8: *K*. *marxianus* αO4-I4, Lanes 9–13: strains not used in this paper. (a) The arrow indicates that the HR-Blank cassette was inserted into the *ALG*3 gene. (b) The arrow indicates that the *GnTII* cassette was inserted into the *KU*70 gene. (c) The arrow indicates that the *MdsI* and *GnTI* cassettes were inserted into the *URA*3 gene. (d) All gene cassettes were inserted into the chromosome and the inserted gene cassettes were validated by PCR, using the S1274 and S1276 primer pairs. The arrows indicate the transformed genes of different fragment sizes. (e) Validation of the *MdsI* gene insertion in the *URA3* gene by PCR with the primer pair: ura3-F and MdsI-788R. (f) Validation of the *Cas9* gene in the cell by PCR with the primer pair: S1274-F and Cas9-M2R. (g) Validation of the mating-types of the transformants by PCR with the primer pair: Haploid-FP1 and Haploid-RP1. The arrow indicates the α type fragment; the other fragment is the a type. If the strain is a diploid, it includes both fragments.
(TIF)

S6 Fig Validation of the knockouts and knockins of donor DNAs to the target gene in antibiotic-free strains by PCR. N: Negative control, M: DNA marker, Lane 1: αO4-I3ΔC, Lane 2: αO4-I4ΔC, Lane 3: αO4-I3ΔR, Lane 4: αO4-I4ΔR. (a) All gene cassettes were inserted to the chromosome and the genes inserted were validated by PCR, using the S1274F and S1276R primer pairs. The white font indicates the different fragment sizes of the transformed genes on the left side of the figure. We used the S1274F and MdsI-R2 primer pairs to confirm the three strains that were supposed to carry by the *MdsI* gene (right side of the figure). (b) The left side of the figure confirmed that the *GnTI* gene was inserted into the *URA3* gene position; it was checked by PCR using the URA3-F and GnTI-R primer pairs. The right side of the figure confirmed that the mating-type was retained on the α haploid. (c) The left side of the figure confirmed that the *MdsI* gene was inserted into the *URA3* gene; it was checked by PCR using the URA3-F and MdsI-R2 primer pairs. The right side of the figure confirmed that the *GnTI* gene was retained on the transformants by PCR using the S1274F and GnTI-R primer pairs. (d) Validation of the *Cas9* gene in the cell by PCR using the primer pair: S1274F and Cas9-M2R (left side of the figure). The white font indicates that *GnTII* was inserted into the *KU70* gene (right side of the figure). (e) Validation of the retention of *G418* in the transformants by PCR using the primer pair: SAD-F1 and G418-R (left side of the figure). Because the PCK protocol was used to knock out the *hygromycin* gene in all strains, no band of *hygromycin* was found in the chromosome by PCR using the primer pair: SAD-F1 and Hyg-R. (f) The *zeocin* gene is adjacent to the *Cas9* gene and it was identified in those transformants carrying the *Cas9* gene.
(TIF)

S7 Fig Plasmid maps of the constructs used in this study. (a) The pMH-1 plasmid includes a signal peptide coding sequence of the *S*. *cerevisiae* α-mating factor and an open reading frame (ORF) of the 1,2-α-mannosidase cloned from *T*. *reesei*. The signal peptide coding sequence of ER reentrant is HDEL and includes a stop codon. (b) The pMH-2 plasmid includes a signal peptide coding sequence of the *Mnn9p* from *S*. *cerevisiae* and an open reading frame of the human β-1,2-*N*-acetylglucosaminyltransferase I. The ORF includes a stop codon and a 12x His-Tag sequence at the end. (c) The pMH-3 plasmid includes the signal peptide coding sequence of the *Mnn2p* from *S*. *cerevisiae* and an open reading frame of the mouse β-1,2-*N*-acetylglucosaminyltransferase II. The ORF includes a stop codon and a 12x His-Ttag at the end.
(TIF)

S8 Fig The proportions of phosphorylated glycans in our transformants. The proportions of phosphorylated glycans are higher in *K*. *marxianus* glycoengineered strains than α2 wild type. (a) *K*. *marxianus* αO3-I2, αO4-I3, αO4-I3ΔC and αO4-I4ΔC were glycoengineered strain. Their phosphorylation is significantly higher than a2. The production of total glycan was increased to 24% in αO4-I3ΔC and 28.4% in αO4-I4ΔC. Phosphorylated glycoforms focus on Man_5-6_GlcNAc_2_. (b) *K*. *marxianus* αO4-I4, αO4-I3ΔR and αO4-I4ΔR were glycoengineered strain. Their phosphorylation is significantly higher than a2. The production of total glycan was increased to 41.6% in αO4-I3ΔR and 36.6% in αO4-I4ΔR. Phosphorylated glycoforms focus on Man_5-6_GlcNAc_2_.
(TIF)

S1 Table The list of all plasmids used in this study. The plasmids (pMH-1 to pMH-3) contained the gene for glycosyltransferase with specialized anchor positioning signal peptides, *LAC4* promoter (P_LAC4_), and terminator, which was constructed in the pU18 vector. The donor DNA PCR was also constructed in the pU18-genes vector. The plasmids (pMH-g1 to pMH-g12) of the gRNA expression cassette contained the SNR52 promoter and the SUP40 terminator.
(DOCX)

S2 Table The list of the yeast strains used in this study. (DOCX)

S3 Table The list of all primer pairs used. These primers were for the construction of gRNA cassettes, homologous recombination of donor DNA cassettes and confirmation of target gene knockout fragments by PCR.
(DOCX)

S1 Raw images (PDF)
