# Supplementary figures and images for: Constructing a human complex type N-linked glycosylation pathway in Kluyveromyces marxianus
Source: PLoS One. 2020 May 29;15(5):e0233492. doi: 10.1371/journal.pone.0233492 (PMC7259728; doi:10.1371/journal.pone.0233492)

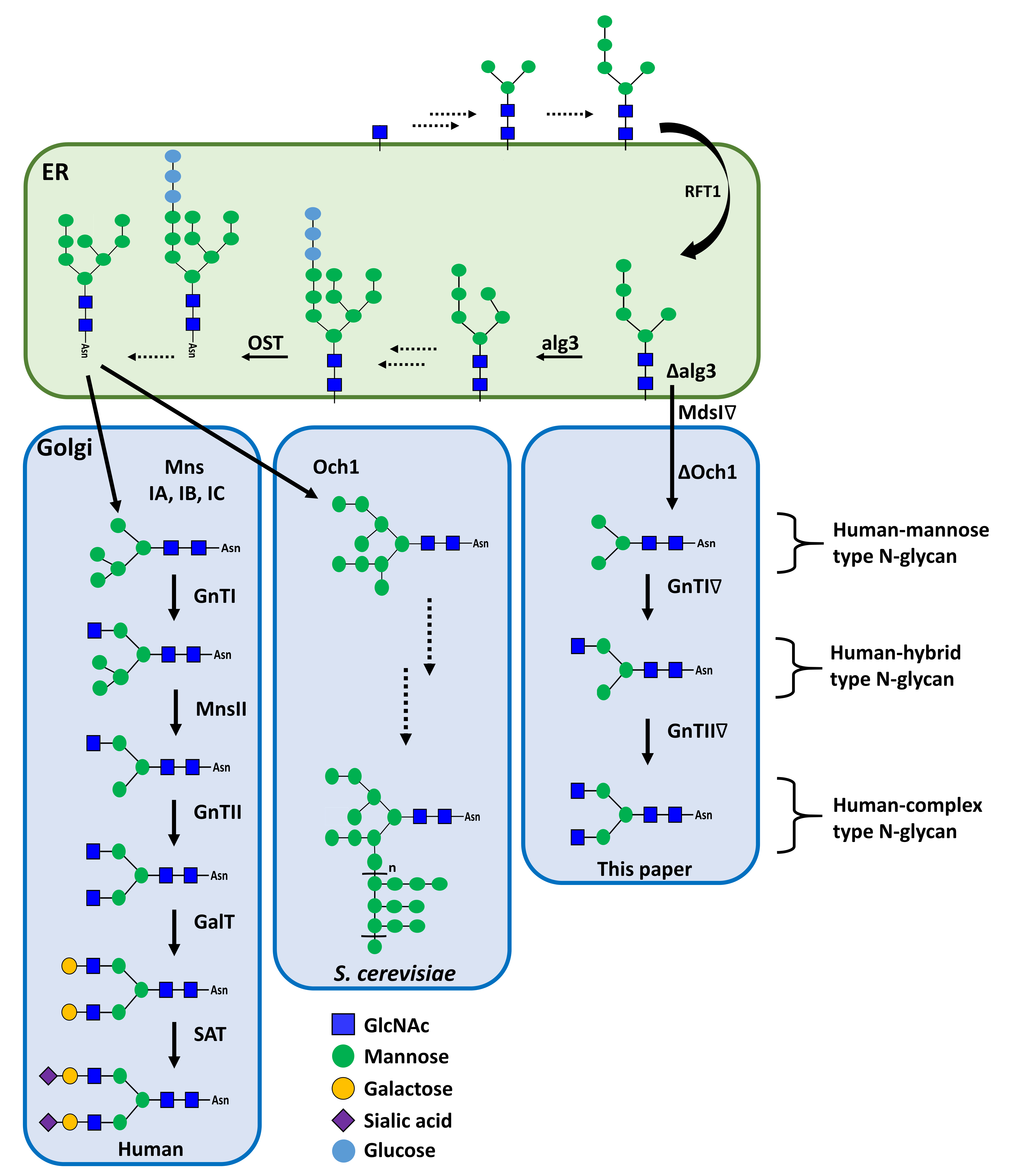

Supplement: S1 Fig — The glycosylation pathway in the ER is the same from yeast to human. The human glycosylation in the Golgi (left panel) requires the following glycosyltransferases [46]: GnTI (β-1,2-N-acetylglucosaminyltransferase I), GnTII (β-1,2-N-acetylglucosaminyltransferase II), GalT (β-1,4-galactosyltransferase I) and ST (sialyltransferase). In S. cerevisiae (middle panel), hypermannosylation is initiated in the Golgi by the α1,6-mannosyltransferase (OCH1), which adds mannoses onto the α1,3 branch of the tri-mannose core, generating an α1,6-linked mannose branch. Additional mannosyltransferases subsequently extend this branch, leading to hypermannosylation. In this study we propose to knock out the ALG3 and OCH1 genes and knock in MdsI (α-1,2-mannosidase), GnTI and GnTII to produce the complex glycoform GlcNAc2Man3GlcNAc2. (TIF) [file pone.0233492.s001.tif]

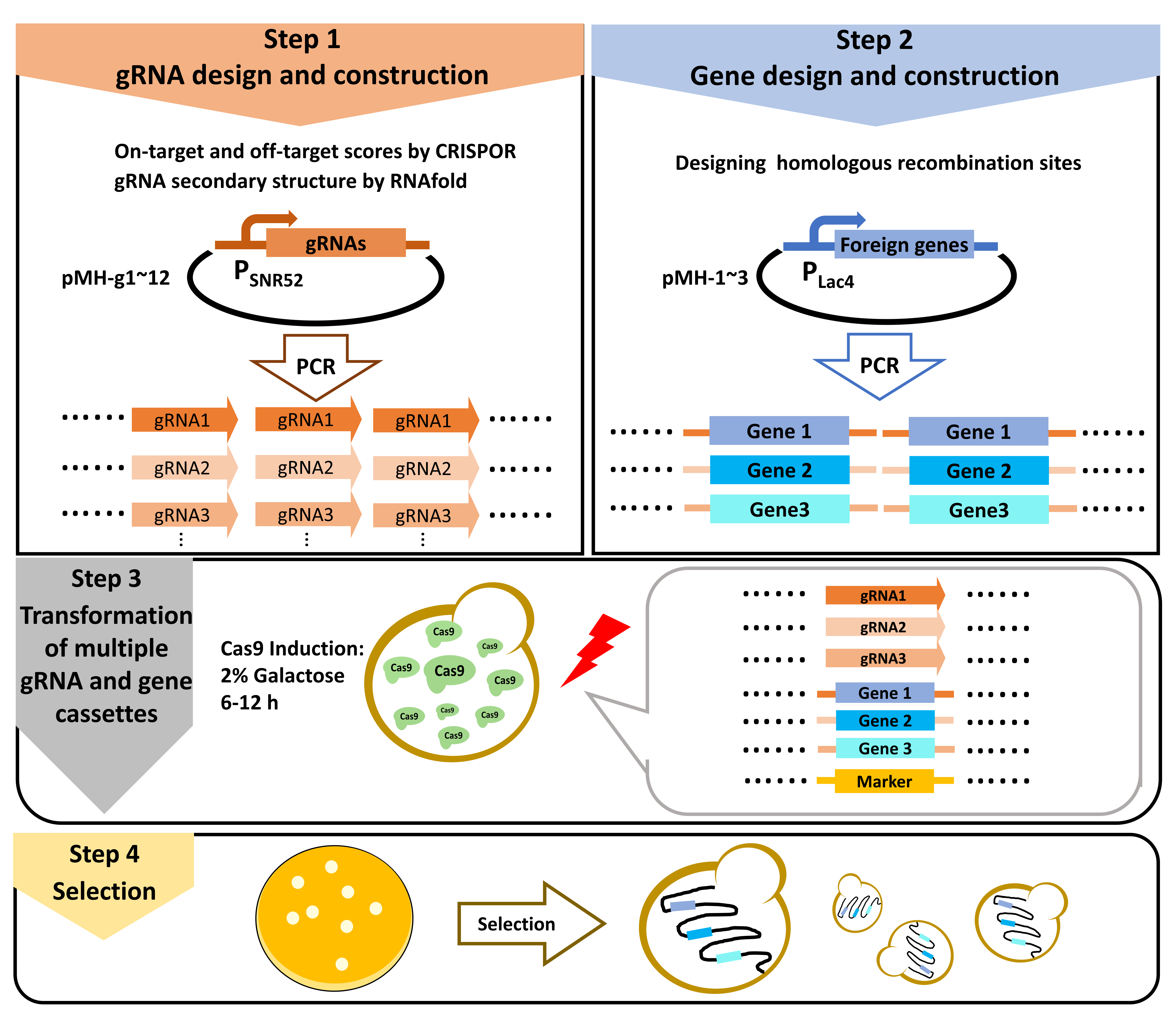

Supplement: S2 Fig — Step 1: gRNA design and construction. To exclude off-targets and improve on-target efficiency, we use the CRISPOR software (http://crispor.tefor.net/). For gRNA secondary structure calculation, we use the bioinformatical tool RNAfold Webserver(http://rna.tbi.univie.ac.at/cgi-bin/RNAWebSuite/RNAfold.cgi). The designed gRNA is constructed on the T&A vector. The double-stranded gRNA expression cassette is amplified by PCR using the M13 primer pairs. Step 2: Gene or donor DNA cassette design and construction. A homologous recombination sequence of ~60 bp is designed at the left and right ends of each gRNA site. The primer pairs of the recombination fragments are ligated to the head and tail positions of the target gene cassette for PCR amplification. Step 3: Transformation of gRNA and gene cassettes. Cas9 gene expression is continued for 6 to 12 hours. Linearized gRNA, donor DNA fragments and a selection marker are transformed into yeast cells by electroporation. Step 4: Colony selection. We select strains from the plate. (TIF) [file pone.0233492.s002.tif]

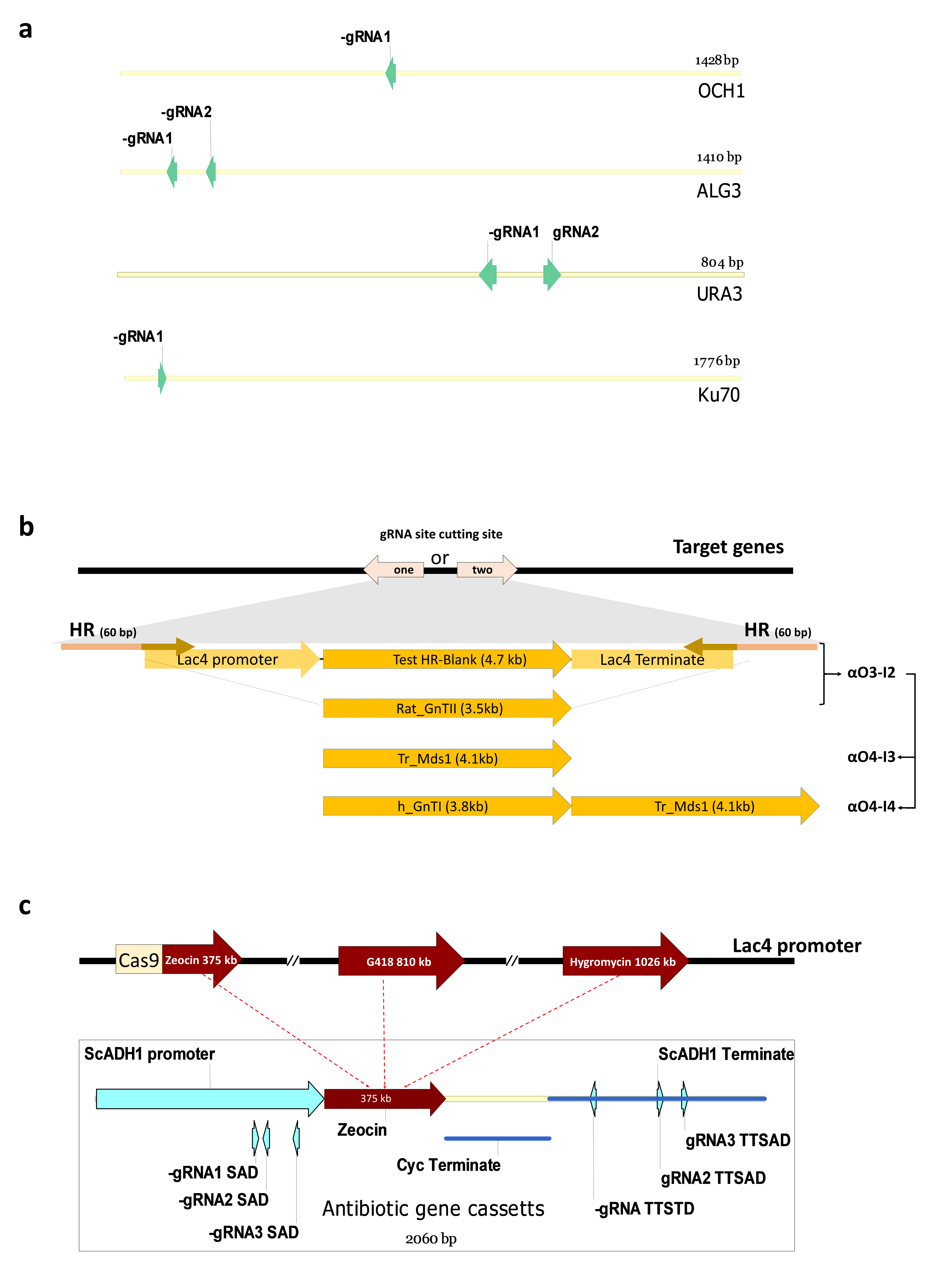

Supplement: S3 Fig — The gRNA cutting sites were also the homologous recombination sites for donor DNA cassettes. (a) The gRNA cutting sites in different target genes. The arrows indicate the gRNA cutting sites. A forward strand DNA is indicated by a right arrow and a reversed strand DNA is indicated by a left arrow. (b) A donor DNA fragment was inserted into the gRNA cutting site in the target gene by homologous recombination. The gray part indicates the gRNA cutting sites of target genes that were also used for the homologous recombination (HR) for the gene expression cassettes. (c) Six gRNA sites were designed in S. cerevisiae PADHI and terminator, which were used for designing antibiotic gene cassettes. Note that the Cas9 coding region is in front of a zeocin cassette and is repeated in the PLAC4 region. When the zeocin cassette is cut, the area of PLAC4 will be rearranged, giving rise a chance to remove the Cas9 gene. (TIF) [file pone.0233492.s003.tif]

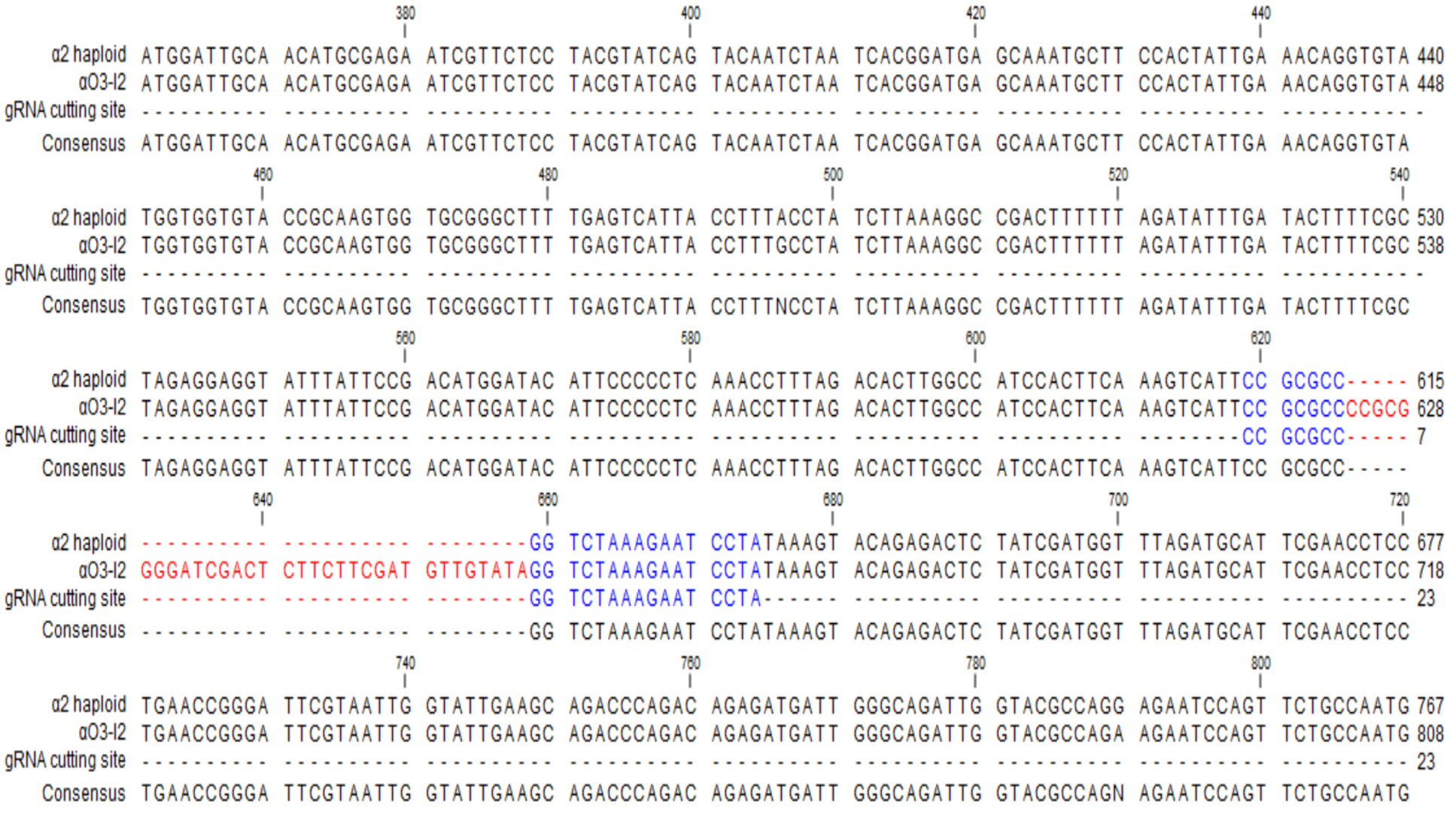

Supplement: S4 Fig — The blue color indicates the original sequence and the red color indicates the regions with insertion or deletion. The αO3-I2 strain contains the 33 bp insertion at the OCH1 gRNA cutting site. (TIF) [file pone.0233492.s004.tif]

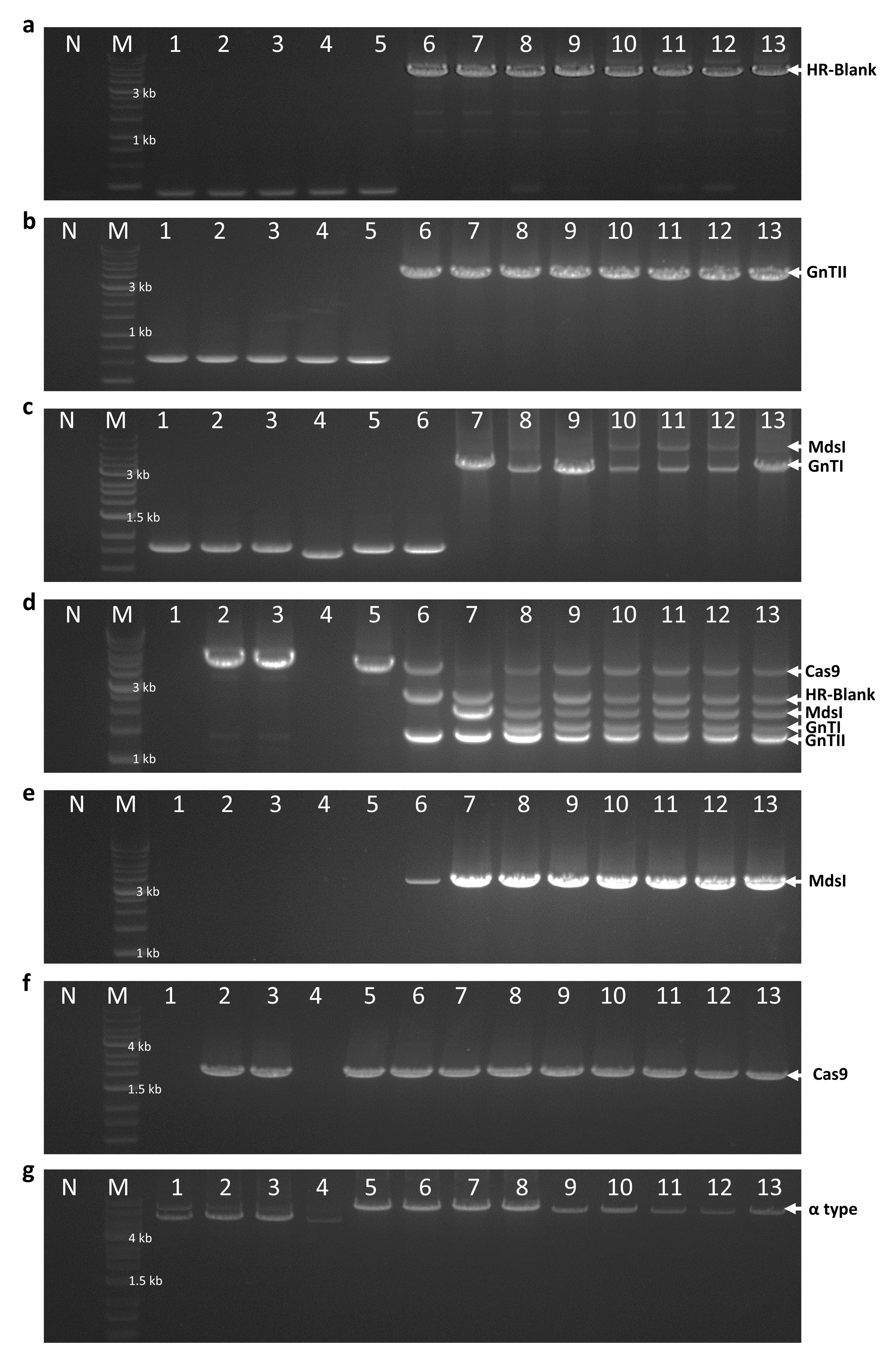

Supplement: S5 Fig — N: negative control; M: DNA marker. Lane 1: the 4G5 wild type, Lanes 2–4: strains not used in this paper; Lane 5: Cas9-carrying K. marxianus α2; Lane 6: K. marxianus αO3-I2, Lane 7: K. marxianus αO4-I3, Lane 8: K. marxianus αO4-I4, Lanes 9–13: strains not used in this paper. (a) The arrow indicates that the HR-Blank cassette was inserted into the ALG3 gene. (b) The arrow indicates that the GnTII cassette was inserted into the KU70 gene. (c) The arrow indicates that the MdsI and GnTI cassettes were inserted into the URA3 gene. (d) All gene cassettes were inserted into the chromosome and the inserted gene cassettes were validated by PCR, using the S1274 and S1276 primer pairs. The arrows indicate the transformed genes of different fragment sizes. (e) Validation of the MdsI gene insertion in the URA3 gene by PCR with the primer pair: ura3-F and MdsI-788R. (f) Validation of the Cas9 gene in the cell by PCR with the primer pair: S1274-F and Cas9-M2R. (g) Validation of the mating-types of the transformants by PCR with the primer pair: Haploid-FP1 and Haploid-RP1. The arrow indicates the α type fragment; the other fragment is the a type. If the strain is a diploid, it includes both fragments. (TIF) [file pone.0233492.s005.tif]

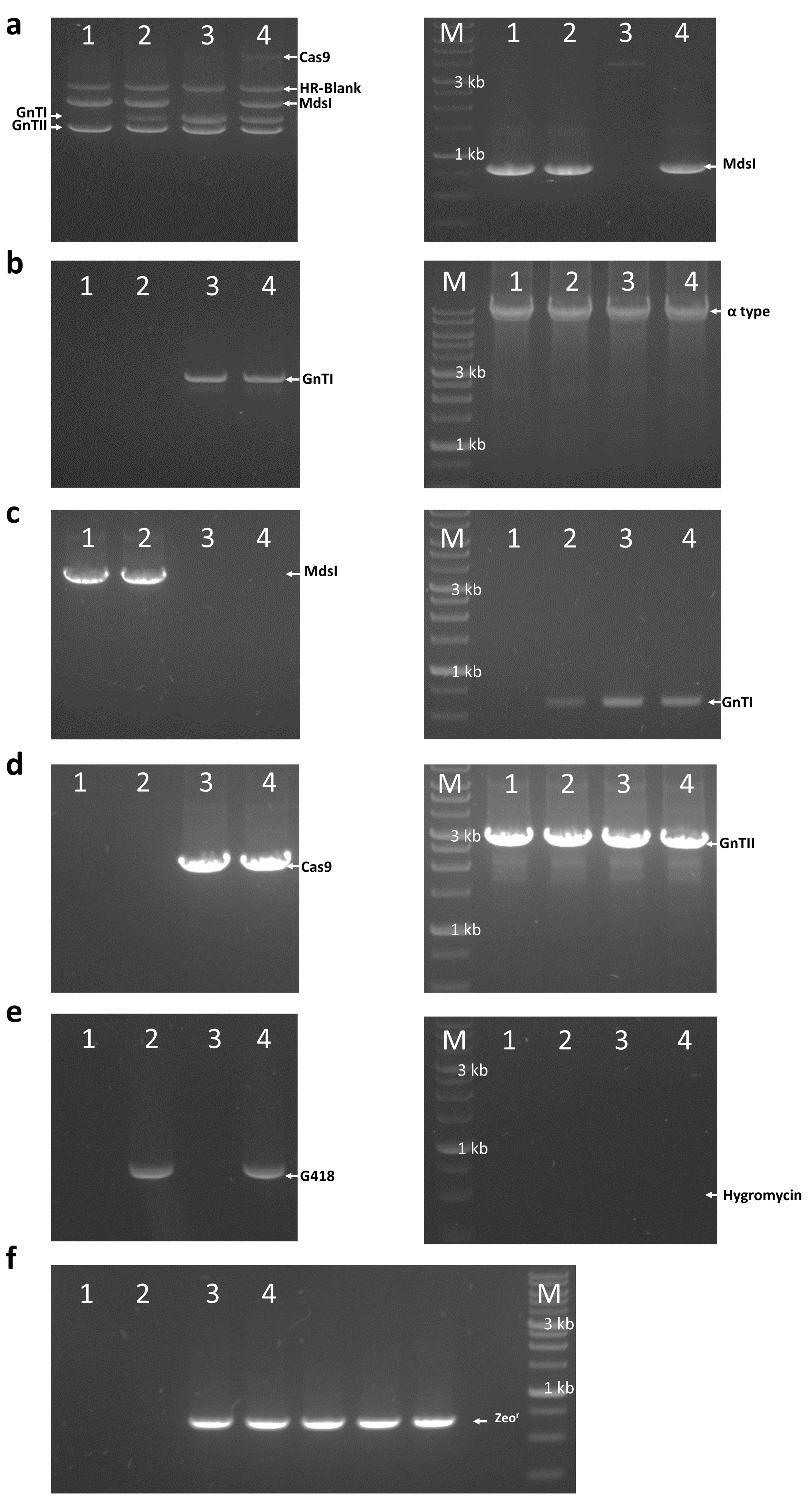

Supplement: S6 Fig — N: Negative control, M: DNA marker, Lane 1: αO4-I3ΔC, Lane 2: αO4-I4ΔC, Lane 3: αO4-I3ΔR, Lane 4: αO4-I4ΔR. (a) All gene cassettes were inserted to the chromosome and the genes inserted were validated by PCR, using the S1274F and S1276R primer pairs. The white font indicates the different fragment sizes of the transformed genes on the left side of the figure. We used the S1274F and MdsI-R2 primer pairs to confirm the three strains that were supposed to carry by the MdsI gene (right side of the figure). (b) The left side of the figure confirmed that the GnTI gene was inserted into the URA3 gene position; it was checked by PCR using the URA3-F and GnTI-R primer pairs. The right side of the figure confirmed that the mating-type was retained on the α haploid. (c) The left side of the figure confirmed that the MdsI gene was inserted into the URA3 gene; it was checked by PCR using the URA3-F and MdsI-R2 primer pairs. The right side of the figure confirmed that the GnTI gene was retained on the transformants by PCR using the S1274F and GnTI-R primer pairs. (d) Validation of the Cas9 gene in the cell by PCR using the primer pair: S1274F and Cas9-M2R (left side of the figure). The white font indicates that GnTII was inserted into the KU70 gene (right side of the figure). (e) Validation of the retention of G418 in the transformants by PCR using the primer pair: SAD-F1 and G418-R (left side of the figure). Because the PCK protocol was used to knock out the hygromycin gene in all strains, no band of hygromycin was found in the chromosome by PCR using the primer pair: SAD-F1 and Hyg-R. (f) The zeocin gene is adjacent to the Cas9 gene and it was identified in those transformants carrying the Cas9 gene. (TIF) [file pone.0233492.s006.tif]

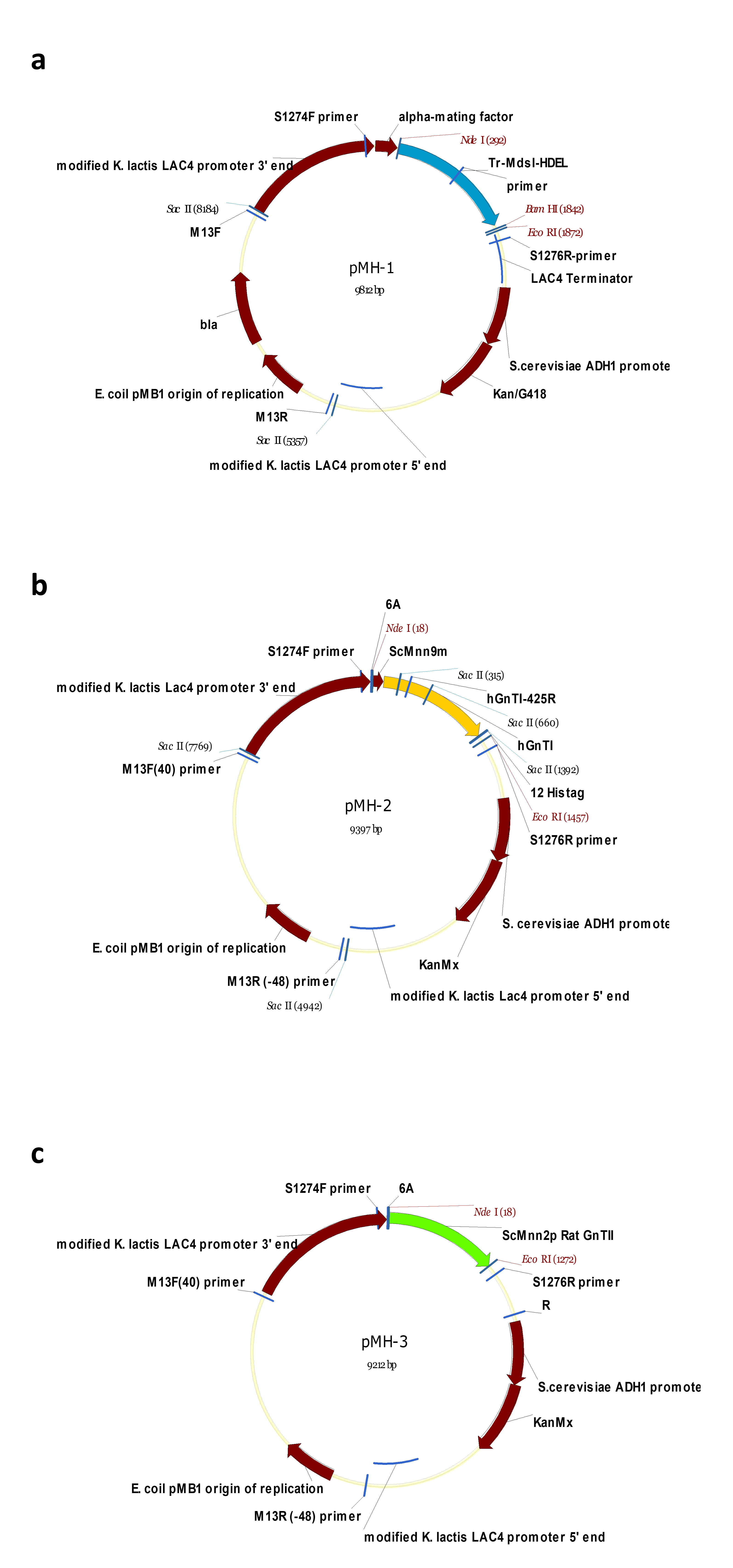

Supplement: S7 Fig — (a) The pMH-1 plasmid includes a signal peptide coding sequence of the S. cerevisiae α-mating factor and an open reading frame (ORF) of the 1,2-α-mannosidase cloned from T. reesei. The signal peptide coding sequence of ER reentrant is HDEL and includes a stop codon. (b) The pMH-2 plasmid includes a signal peptide coding sequence of the Mnn9p from S. cerevisiae and an open reading frame of the human β-1,2-N-acetylglucosaminyltransferase I. The ORF includes a stop codon and a 12x His-Tag sequence at the end. (c) The pMH-3 plasmid includes the signal peptide coding sequence of the Mnn2p from S. cerevisiae and an open reading frame of the mouse β-1,2-N-acetylglucosaminyltransferase II. The ORF includes a stop codon and a 12x His-Ttag at the end. (TIF) [file pone.0233492.s007.tif]

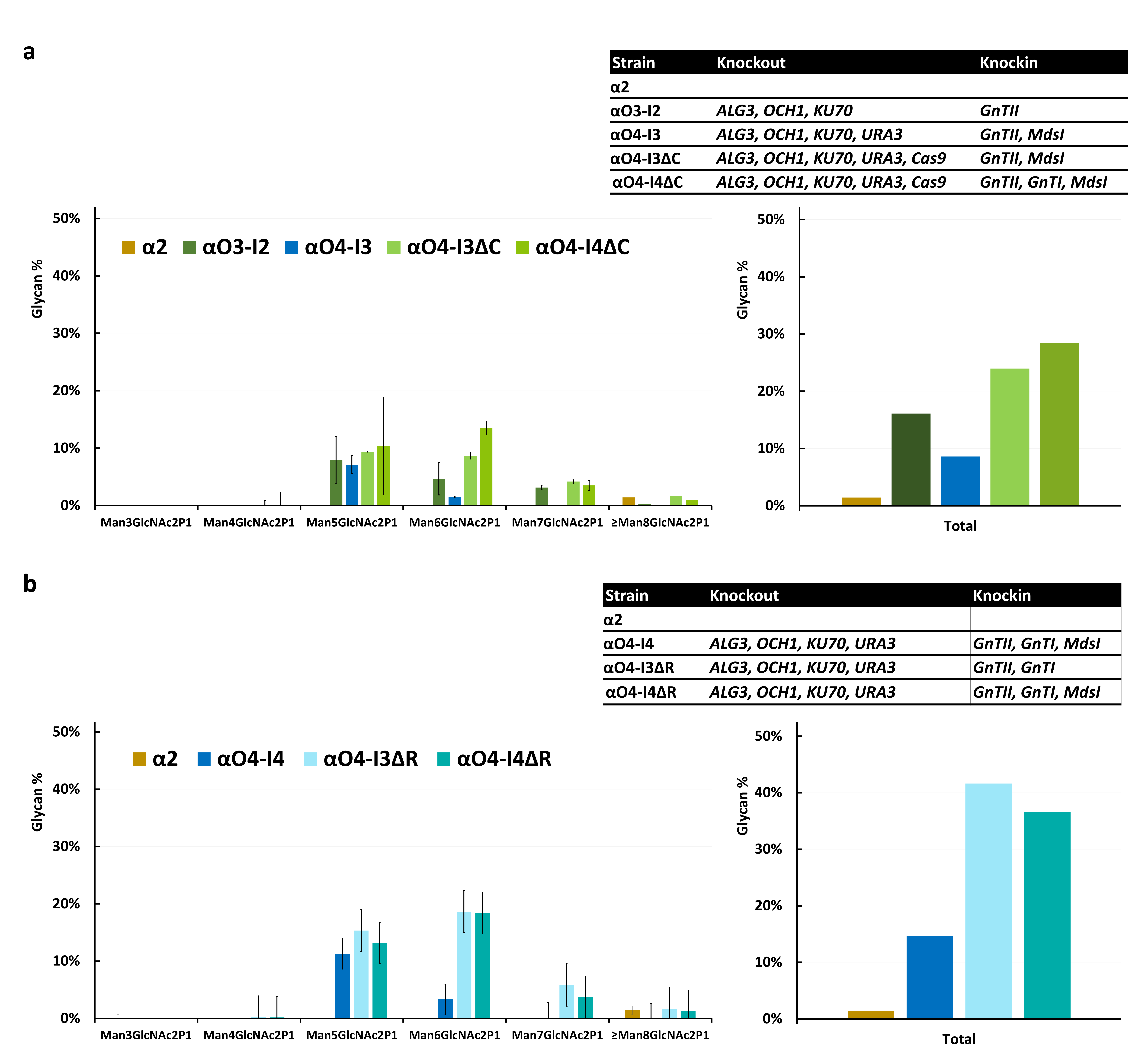

Supplement: S8 Fig — The proportions of phosphorylated glycans are higher in K. marxianus glycoengineered strains than α2 wild type. (a) K. marxianus αO3-I2, αO4-I3, αO4-I3ΔC and αO4-I4ΔC were glycoengineered strain. Their phosphorylation is significantly higher than a2. The production of total glycan was increased to 24% in αO4-I3ΔC and 28.4% in αO4-I4ΔC. Phosphorylated glycoforms focus on Man5-6GlcNAc2. (b) K. marxianus αO4-I4, αO4-I3ΔR and αO4-I4ΔR were glycoengineered strain. Their phosphorylation is significantly higher than a2. The production of total glycan was increased to 41.6% in αO4-I3ΔR and 36.6% in αO4-I4ΔR. Phosphorylated glycoforms focus on Man5-6GlcNAc2. (TIF) [file pone.0233492.s008.tif]
