# Supplementary material for: Constructing a human complex type N-linked glycosylation pathway in Kluyveromyces marxianus
Source: PLoS One. 2020 May 29;15(5):e0233492. doi: 10.1371/journal.pone.0233492 (PMC7259728; doi:10.1371/journal.pone.0233492)
Supplement: S1 Table — The plasmids (pMH-1 to pMH-3) contained the gene for glycosyltransferase with specialized anchor positioning signal peptides, LAC4 promoter (PLAC4), and terminator, which was constructed in the pU18 vector. The donor DNA PCR was also constructed in the pU18-genes vector. The plasmids (pMH-g1 to pMH-g12) of the gRNA expression cassette contained the SNR52 promoter and the SUP40 terminator. (DOCX) [file pone.0233492.s009.docx]

**S1 Table. The list of all plasmids used in this study.** The plasmids (pMH-1 to pMH-3) contained the gene for glycosyltransferase with specialized anchor positioning signal peptides, pKLac4 promoter, and terminator, which was constructed in the pU18 vector. The donor DNA PCR was also constructed in the pU18-genes vector. The plasmids (pMH-g1 to pMH-g12) of the gRNA expression cassette contained the SNR52 promoter and the SUP40 terminator.

| **Plasmid name** | **Signal peptide** | **Promoter** | **Gene name (**∆a.a.**)** | **Vector** |
| --- | --- | --- | --- | --- |
| pMH-1 | α-factor | pKlac4 | MdsI (∆185) | pKLac4, pU18 |
| pMH-2 | ScMnn9p | pKlac4 | hGnTI (∆43) | pKLac4, pU18 |
| pMH-3 | ScMnn2p | pKlac4 | RatGnTII (∆88) | pKLac4, pU18 |
| **Plasmid name** | **gRNA sequence** | **Promoter** | **gRNA name** | **Vector** |
| pMH-g1 | TGCAAAATGCAATACCTTCGTGG | SNR52 | Ku70 gRNA | T&A |
| pMH-g2 | GCGCACGGTGAATACACTCGTGG | SNR52 | Ura3 gRNA1 | T&A |
| pMH-g3 | ATTGCTCAAAACGATATGGGTGG | SNR52 | Ura3 gRNA2 | T&A |
| pMH-g4 | TCTTGCAATGGTGTGAATGGTGG | SNR52 | Alg3 gRNA1 | T&A |
| pMH-g5 | TGGCTAACGACTCCAGTATGAGG | SNR52 | Alg3 gRNA2 | T&A |
| pMH-g6 | TAGGATTCTTTAGACCGGCGCGG | SNR52 | Och1 gRNA | T&A |
| pMH-g7 | CACTACTCTCTAATGAGCAA | SNR52 | SAD gRNA1 | T&A |
| pMH-g8 | AATTCAAGTAACTGGAAGGA | SNR52 | SAD gRNA2 | T&A |
| pMH-g9 | AGGGAACGAGAACAATGACG | SNR52 | SAD gRNA3 | T&A |
| pMH-g10 | GTAATCTAGATCGACCAAAG | SNR52 | TTSAD gRNA1 | T&A |
| pMH-g11 | ACTTTTTATACATAGAACAT | SNR52 | TTSAD gRNA2 | T&A |
| pMH-g12 | TTAGACAAGTTGTTGAGAAC | SNR52 | TTSAD gRNA3 | T&A |
