# Supplementary material for: Constructing a human complex type N-linked glycosylation pathway in Kluyveromyces marxianus
Source: PLoS One. 2020 May 29;15(5):e0233492. doi: 10.1371/journal.pone.0233492 (PMC7259728; doi:10.1371/journal.pone.0233492)
Supplement: S2 Table — (DOCX) [file pone.0233492.s010.docx]

**S2 Table. The list of the yeast strains used in this study.**

| **Strain name** | **Genotype** |
| --- | --- |
| ***K. marxianus*** 4G5 (wild type) | *MATα/a* |
| ***K. marxianus*** α2 (wild type) | *MATα*, ∆*Matα3, Lac4::Cas9-Zeo, Lac4:: hyg* |
| ***K. marxianus*** αO3-I2 | *MATα*, ∆*Matα3*, *Lac4::Cas9-Zeo, Lac4::hyg, Lac4::G418, och1*::(+33bp), *ku70*::*GnTII*, *alg3*::HR-Blank |
| ***K. marxianus*** αO4-I3 | *MATα*, ∆*Matα3*, *Lac4::Cas9-Zeo, Lac4::hyg, Lac4::G418, och1*::(+33bp), *ku70*::*GnTII*, *alg3*::HR-Blank, *ura3*::MdsI |
| ***K. marxianus*** αO4-I4 | *MATα*, ∆*Matα3*, *Lac4::Cas9-Zeo, Lac4::hyg, Lac4::G418, och1*::(+33bp), *ku70*::*GnTII*, *alg3*::HR-Blank, *ura3*::*MdsI*-*GnTI* |
| ***K. marxianus*** αO4-I3∆C | *MATα*, ∆*Matα3*, *och1*::(+33bp), *ku70*::*GnTII*, *alg3*::HR-Blank, *ura3*::*MdsI* |
| ***K. marxianus*** αO4-I4∆C | *MATα*, ∆*Matα3*, *och1*::(+33bp), *ku70*::*GnTII*, *alg3*::HR-Blank, *ura3*::*MdsI, Lac4::GnTI-G418* |
| ***K. marxianus*** αO4-I4∆R | *MATα*, ∆*Matα3*, *Lac4::Cas9-Zeo, och1*::(+33bp), *ku70*::*GnTII*, *alg3*::HR-Blank, *ura3*::*MdsI, Lac4::MdsI-G418* |
| ***S. cerevisiae* BY4741** (wild type) | *MATa*, *his3*Δ1, *leu2*Δ0, *met15*Δ0, *ura3*Δ0 |
| ***K. lastis KB101*** (wild type) | *MATa*, Δ*ade*, Δ*trpl*, *ura3*::*gal80*-1 |
