# Supplementary material for: Constructing a human complex type N-linked glycosylation pathway in Kluyveromyces marxianus
Source: PLoS One. 2020 May 29;15(5):e0233492. doi: 10.1371/journal.pone.0233492 (PMC7259728; doi:10.1371/journal.pone.0233492)
Supplement: S3 Table — These primers were for the construction of gRNA cassettes, homologous recombination of donor DNA cassettes and confirmation of target gene knockout fragments by PCR. (DOCX) [file pone.0233492.s011.docx]

**S3 Table. The list of all primer pairs used.** These primers were for the construction of gRNA cassettes, homologous recombination of donor DNA cassettes and confirmation of target gene knockout fragments by PCR.

| Primers | Sequence (5’-3’) |
| --- | --- |
| och1-gRNA-PR | CGCCGGTCTAAAGAATCCTAGATCATTTATCTTTC |
| och1-gRNA -FT | TAGGATTCTTTAGACCGGCGgttttagagctagaa |
| alg3-gRNA1-PR | CCATTCACACCATTGCAAGAGATCATTTATCTTTC |
| alg3-gRNA1-FT | TCTTGCAATGGTGTGAATGGgttttagagctagaa |
| alg3-gRNA2-PR | CATACTGGAGTCGTTAGCCAGATCATTTATCTTTC |
| alg3-gRNA2-FT | TGGCTAACGACTCCAGTATGgttttagagctagaa |
| ku70-gRNA1-FT | CGAAGGTATTGCATTTTGCAGATCATTTATCTTTC |
| ku70-gRNA1-PR | TGCAAAATGCAATACCTTCGgttttagagctagaa |
| ura3-gRNA1-PR | AGGGGTCTCTAGCGCACGGTGATCATTTATCTTTC |
| ura3-gRNA1-FT | ACCGTGCGCTAGAGACCCCTgttttagagct |
| ura3-gRNA2-PR | CCCATATCGTTTTGAGCAATGATCATTTATCTTTC |
| ura3-gRNA2-FT | ATTGCTCAAAACGATATGGGgttttagagctagaa |
| SAD-gRNA1-PR | GCTATTTCTAGCTCTAAAACTTGCTCATTAGAGAGTAGTGGATCATTTATCTTTCACTGC |
| SAD-gRNA1-FT | gcagtgaaagataaatgatcCACTACTCTCTAATGAGCAAgttttagagctagaaatagc |
| SAD-gRNA2-PR | GCTATTTCTAGCTCTAAAACTCCTTCCAGTTACTTGAATTGATCATTTATCTTTCACTGC |
| SAD-gRNA2-FT | gcagtgaaagataaatgatcAATTCAAGTAACTGGAAGGAgttttagagctagaaatagc |
| SAD-gRNA3-PR | GCTATTTCTAGCTCTAAAACCGTCATTGTTCTCGTTCCCTGATCATTTATCTTTCACTGC |
| SAD-gRNA3-FT | gcagtgaaagataaatgatcAGGGAACGAGAACAATGACGgttttagagctagaaatagc |
| TTSAD-gRNA1-PR | GCTATTTCTAGCTCTAAAACCTTTGGTCGATCTAGATTACGATCATTTATCTTTCACTGC |
| TTSAD-gRNA1-FT | gcagtgaaagataaatgatcGTAATCTAGATCGACCAAAGgttttagagctagaaatagc |
| TTSAD-gRNA2-PR | GCTATTTCTAGCTCTAAAACATGTTCTATGTATAAAAAGTGATCATTTATCTTTCACTGC |
| TTSAD-gRNA2-FT | gcagtgaaagataaatgatcACTTTTTATACATAGAACATgttttagagctagaaatagc |
| TTSAD-gRNA3-PR | GCTATTTCTAGCTCTAAAACGTTCTCAACAACTTGTCTAAGATCATTTATCTTTCACTGC |
| TTSAD-gRNA3-FT | gcagtgaaagataaatgatcTTAGACAAGTTGTTGAGAACgttttagagctagaaatagc |
| alg3HR55_Lac4P25-80F | tggaagatacagtgtctgatgggatgaacaaagaagaaaagtttgaacgaccacc accgcggggatcgactcataaaata |
| alg3HR56-Lac4T24-80R | TCGATCTCGGTGTAGGAAACCGCTTTTTGAATAATTTTCATGGCTAACGACTCCAGTATACAACATCGAAGAAGAGTCTT |
| ku70HR54_Lac4P25-80F | tagtaatgtagggaataaagaccaagagaaatggaaaagatatgaagcccacga accgcggggatcgactcataaaata |
| ku70HR59-Lac4T24-82R | TCAAATCTTCGCTGGGAAGATACATCGATGGGGTTAGCTCAATGCAAAATGCAATACCT ATACAACATCGAAGAAGAGTCTT |
| Ug2HR53-Lac4P24-77F | agttacgaaagaacctagagggttgttaatgcttgccgagttatcgtccaaggCCGCGGGGATCGACTCATAAAATA |
| Ug4HR57-Lac4T24-81R | CAAGACCAACACCTGGCGTCATGATCAACCAATCGTAGCCCTCTTCTCTTCCACCCATATACAACATCGAAGAAGAGTCTT |
| S1274F | GCGGATAACAAGCTCAAC |
| S1276R | TCGGCACTAATAACCGTT |
| alg3(g1)-25F | TGTCTGATGGGATGAACAAAGAAGA |
| alg3(g2)-25R | ATCGATCTCGGTGTAGGAAACCGCT |
| och1-25F | ATGGGAGTTCCAAGAATCTCTAGGA |
| och1-875R | AACTCTTCAACAATGTCAGCGTCAT |
| ku70-F | ATGTCTGATCAAAAACCGGACTTT |
| ku70-571R | ATCTCGAACGGCTTTCTACATCTTG |
| ura3-F | ATGTCGACTAAGAGTTACTCGGAAA |
| ura3-804R | TTAAGCGGATCTGCCTACTCTCTTC |
| MdsI-788R | TTGTAGTGAGCGAATGCAACTGGGT |
| MdsI-R2 | CATTAGCCAAAGTCTGGGCCTGTCTC |
| Cas9-M2R | TTCCTCGATCATCTCTCTGTCCTCA |
| GnTI-R | AGTCTGGTTGTCTGATGTGAGTAAC |
| SAD-F1 | gcaccaacagatgtcgttgttccag |
| G418-R | TACAAACAGGAATCGAATGC |
| Hyg-R | gcctgcgcgacggacgcact |
| Zeo-R | TCAGTCCTGCTCCTCGGCCA |
| Haploid-FP1 | TATACATGGGATCATAAATC |
| Haploid-RP1 | CTTTGTCTTGTATGATATC |
| GnTI-Left Primer | ccaattcagaggtagaagagttcac |
| GnTI-Reft Primer | tggatcgtaaccttcccaag |
| MdsI-Left Primer | tgggttgactccgttacagg |
| MdsI-Right Primer | gaaaccggctgaggagtaga |
| GnTII-Left Primer | aaagttcccaatggctgcta |
| GnTII-Right Primer | ctgatgtcaccccaaccac |
