# Supplementary material for: Constructing a human complex type N-linked glycosylation pathway in Kluyveromyces marxianus
Source: PLoS One. 2020 May 29;15(5):e0233492. doi: 10.1371/journal.pone.0233492 (PMC7259728; doi:10.1371/journal.pone.0233492)
Supplement: S1 Raw images — (PDF) [file pone.0233492.s012.pdf]

Constructing a human complex type N-linked glycosylation pathway in *Kluyveromyces marxianus*

Ming-Hsuan Lee[1,2,3], Tsui-Ling Hsu[4], Jinn-Jy Lin[3], Yu-Ju Lin[3], Yi-Ying Kao[3], Jui-Jen Chang[5] and Wen-Hsiung Li[1,3,6,*]

[1] Doctoral Degree Program in Marine Biotechnology, National Sun Yat-sen University, 70 Lien-Hai Rd., Kaohsiung, 80424, Taiwan

[2]Doctoral Degree Program in Marine Biotechnology, Academia Sinica, 128 Academia Road, Section 2, Nankang, Taipei, 11529, Taiwan

[3]Biodiversity Research Center, Academia Sinica, 128 Academia Road, Sec. 2, Nankang, Taipei 11529, Taiwan

[4] Genomics Research Center, Academia Sinica, 128 Academia Road, Sec. 2, Nankang, Taipei 11529, Taiwan

[5]Department of Medical Research, China Medical University Hospital, China Medical University, No. 91 Hsueh-Shih Road, Taichung 40402, Taiwan

[6]Department of Ecology and Evolution, University of Chicago, Chicago 60637, USA

*Corresponding author: whli@uchicago.edu

Supplementary raw data:

The raw figures used in making Fig 4. and Fig.5 western related figures.

The raw figures used in making Supplementary S5_Fig and S6_Fig related Supplementary Figures.

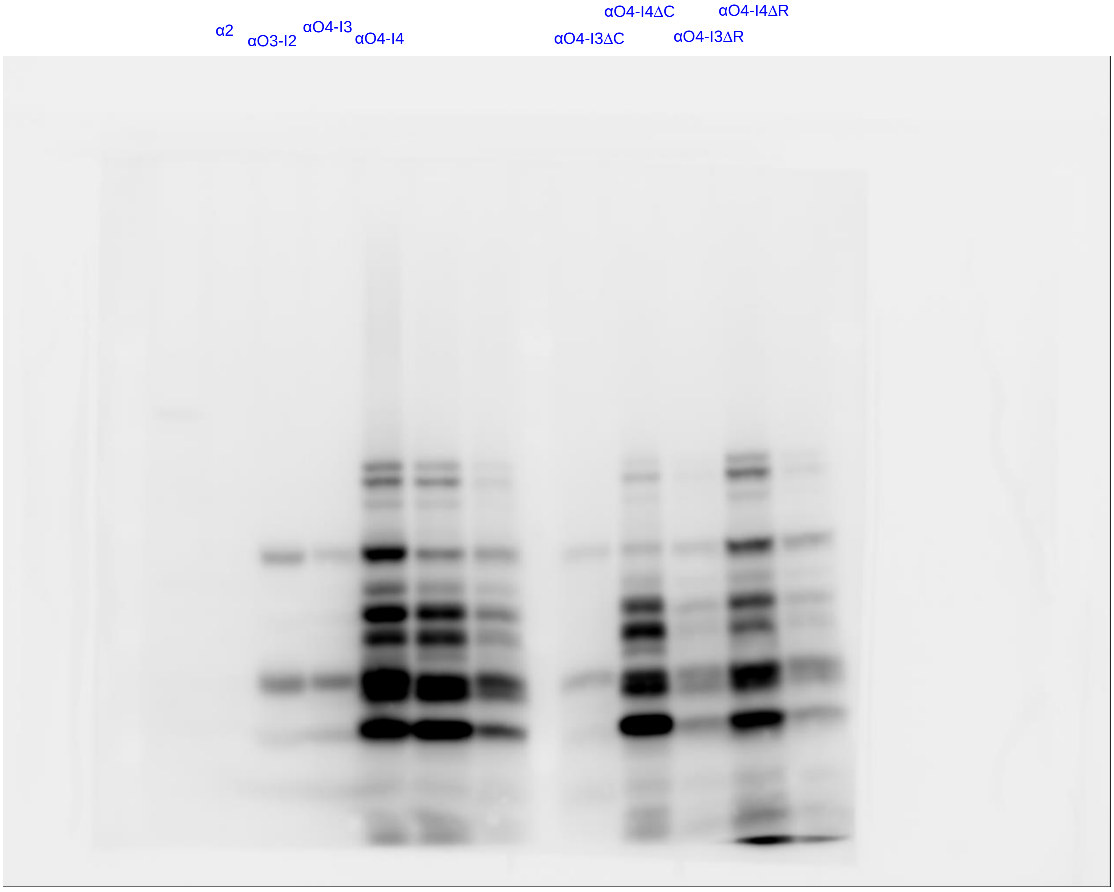

Fig 4. and Fig 5. Analyses of protein expression level and N-glycan profile in K. marxianus α2, αO3-I2, αO4-I3, αO4-I3ΔC, αO4-I4ΔC, αO4-I4, αO4-I3ΔR and αO4-I4ΔR.

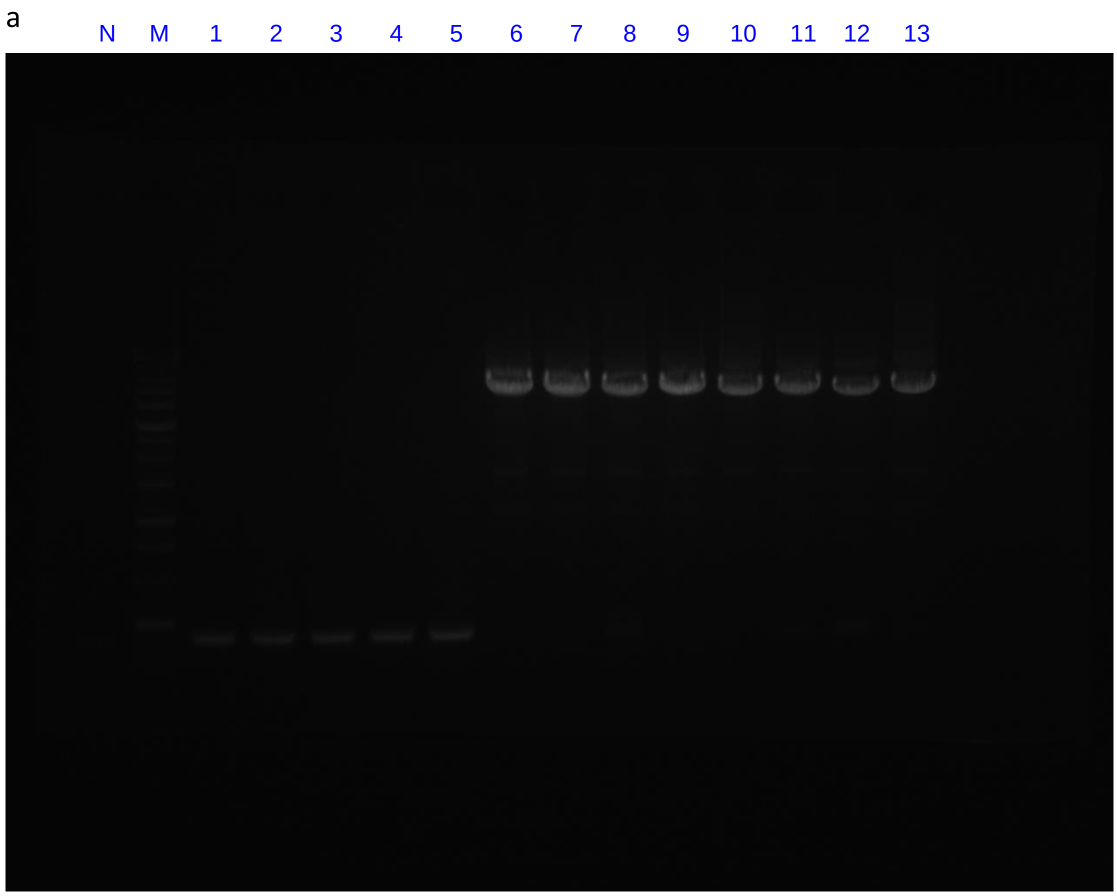
a
N M 1 2 3 4 5 6 7 8 9 10 11 12 13

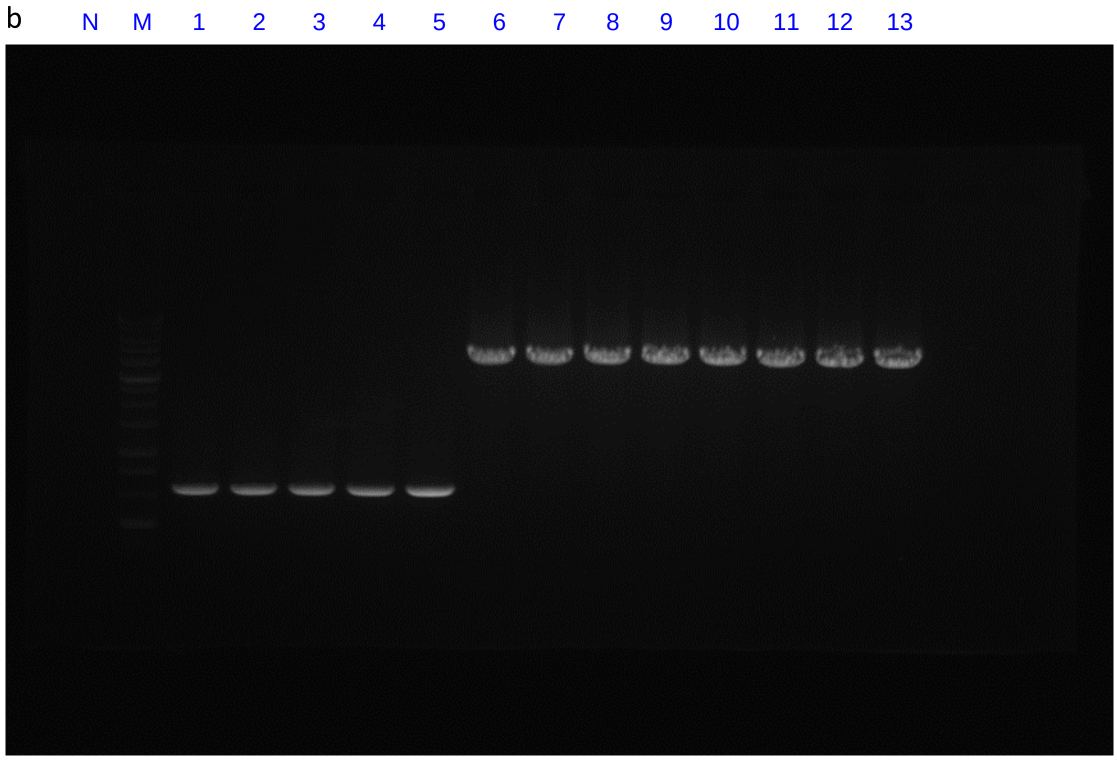
b
N M 1 2 3 4 5 6 7 8 9 10 11 12 13

c

N M 1 2 3 4 5 6 7 8 9 10 11 12 13

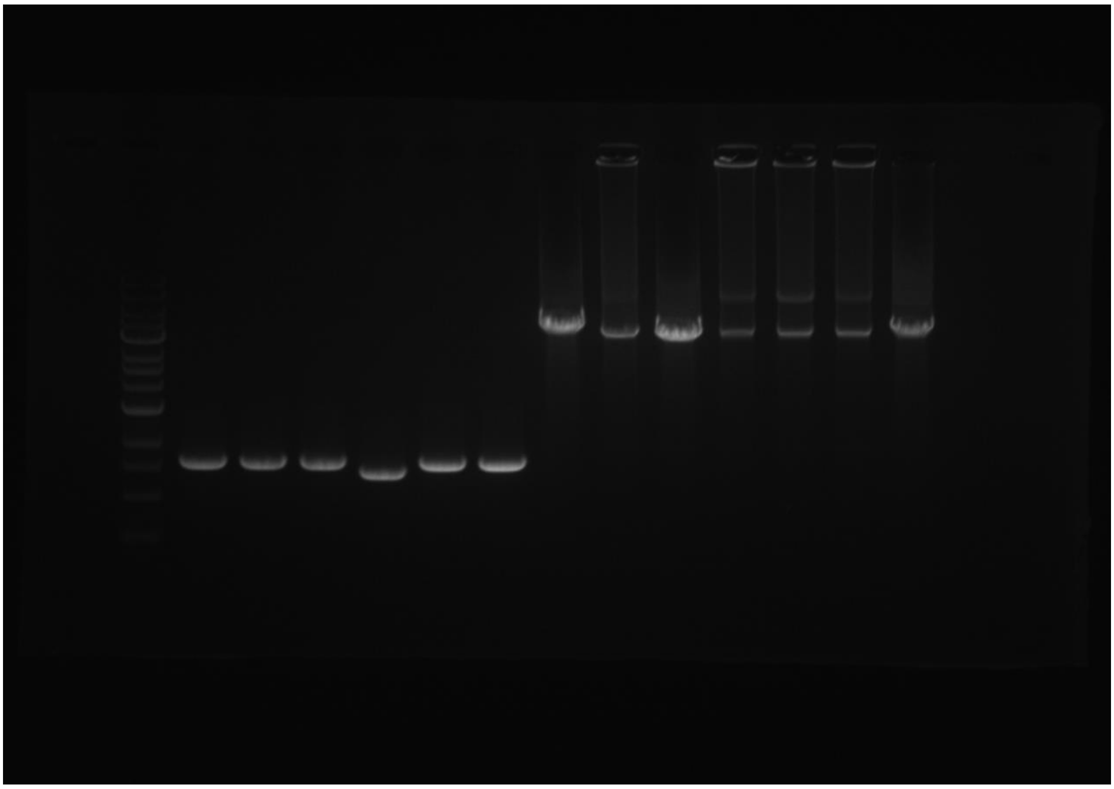

d

N M 1 2 3 4 5 6 7 8 9 10 11 12 13

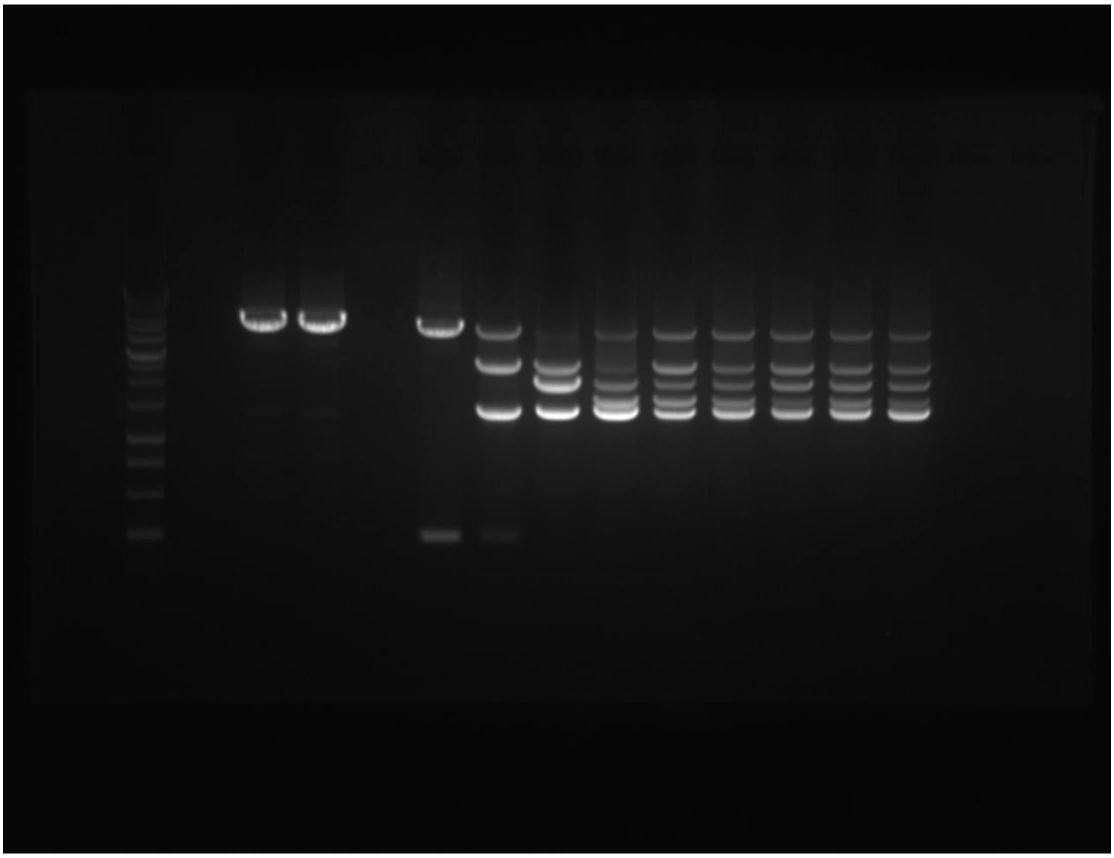

e

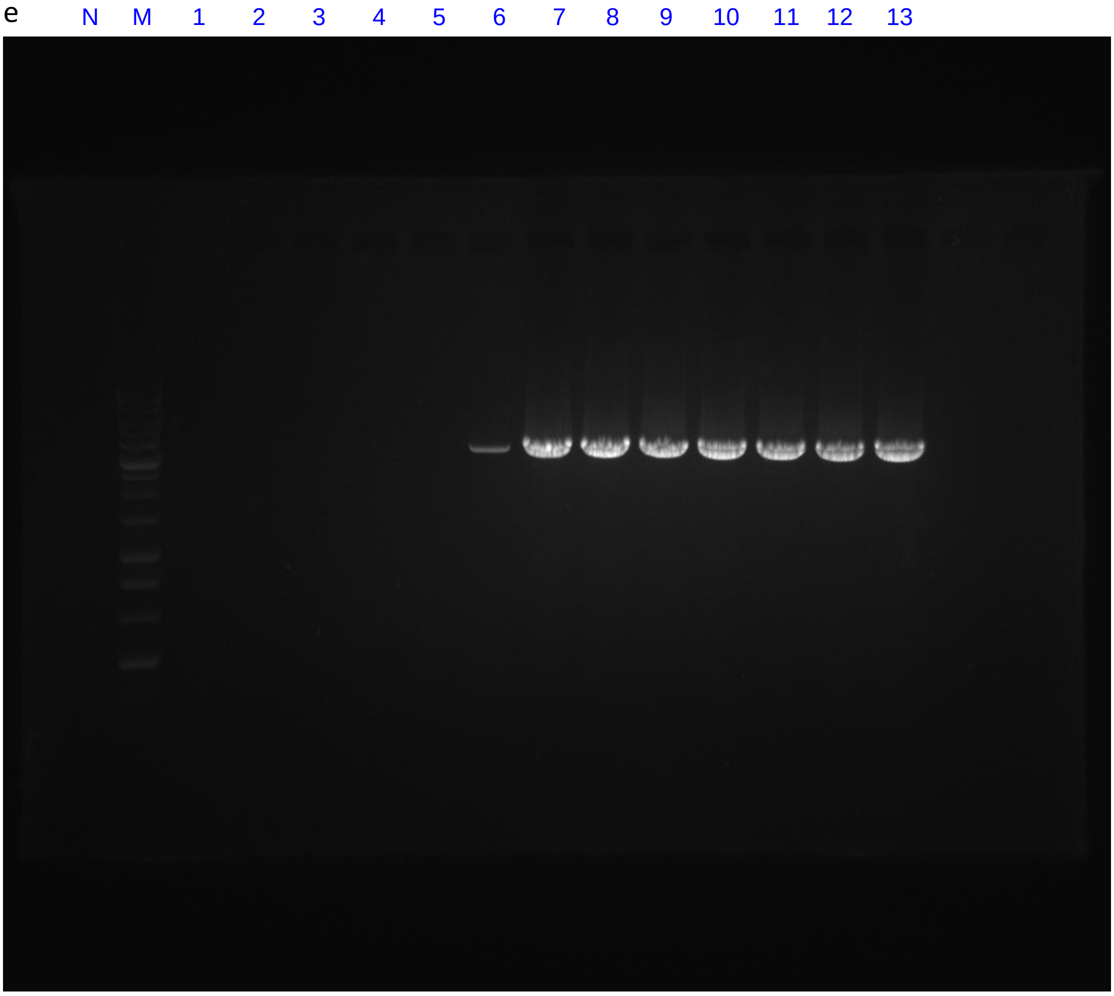

f

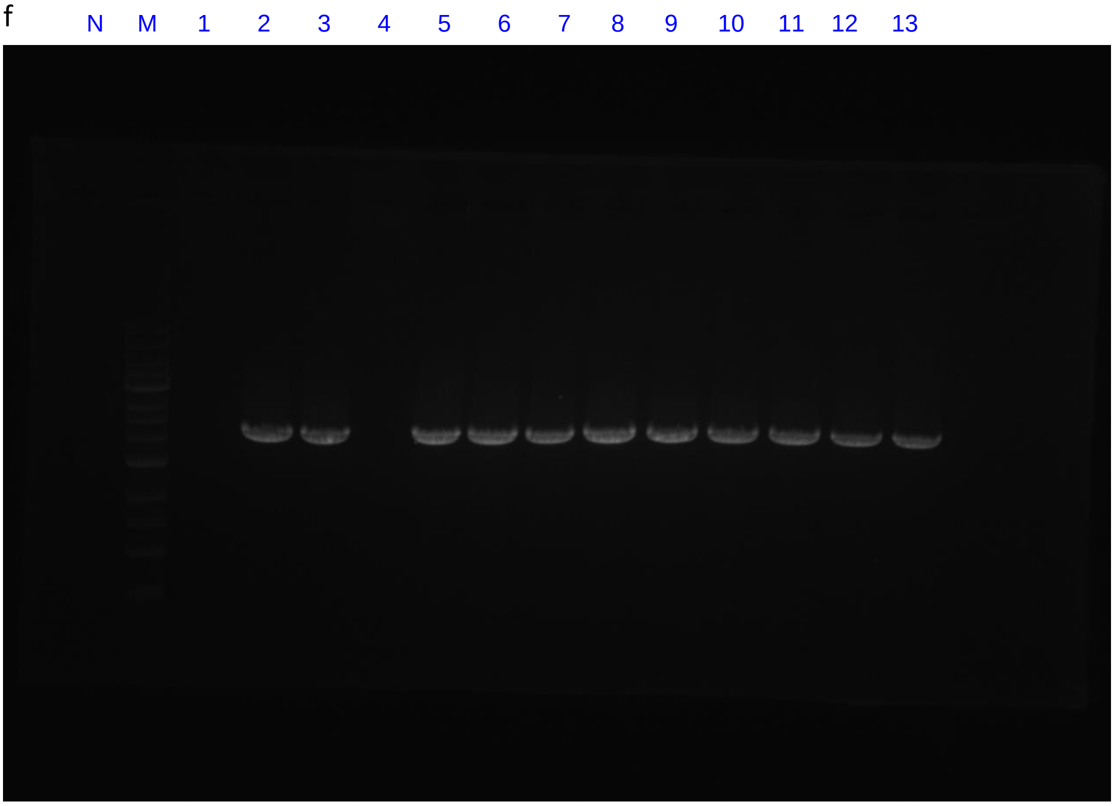

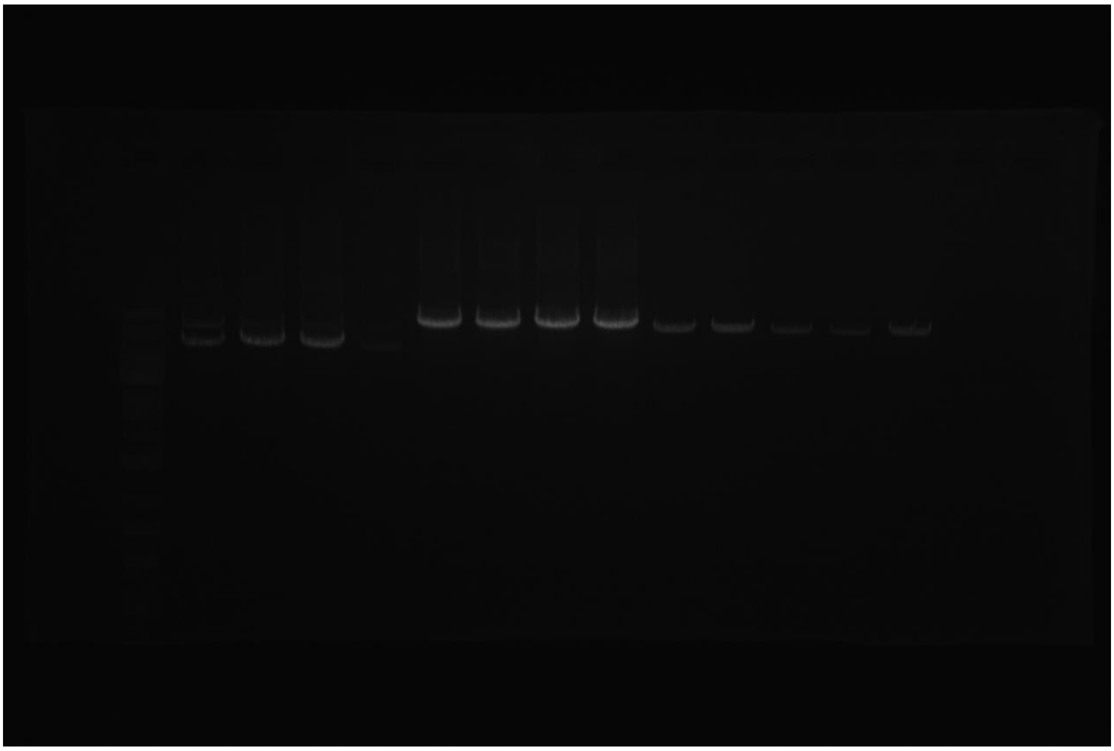

S5 Fig. Validation of the insertions of donor DNAs in transformants by PCR. N: negative control; M: DNA marker. Lane 1: the 4G5 wild type, Lanes 2 - 4: strains not used in this paper; Lane 5: Cas9-carrying *K. marxianus* α2; Lane 6: *K. marxianus* αO3-I2, Lane 7: *K. marxianus* αO4-I3, Lane 8: *K. marxianus* αO4-I4, Lanes 9 - 13: strains not used in this paper. (a) The arrow indicates that the HR-Blank cassette was inserted into the *ALG*3 gene. (b) The arrow indicates that the *GnTII* cassette was inserted into the *KU*70 gene. (c) The arrow indicates that the *MdsI* and *GnTI* cassettes were inserted into the *URA*3 gene. (d) All gene cassettes were inserted into the chromosome and the inserted gene cassettes were validated by PCR, using the S1274 and S1276 primer pairs. The arrows indicate the transformed genes of different fragment sizes. (e) Validation of the *MdsI* gene insertion in the *URA3* gene by PCR with the primer pair: ura3-F and

MdsI-788R. (f) Validation of the *Cas9* gene in the cell by PCR with the primer pair: S1274-F and Cas9-M2R. (g) Validation of the mating-types of the transformants by PCR with the primer pair: Haploid-FP1 and Haploid-RP1. The arrow indicates the α type fragment; the other fragment is the a type. If the strain is a diploid, it includes both fragments.

a

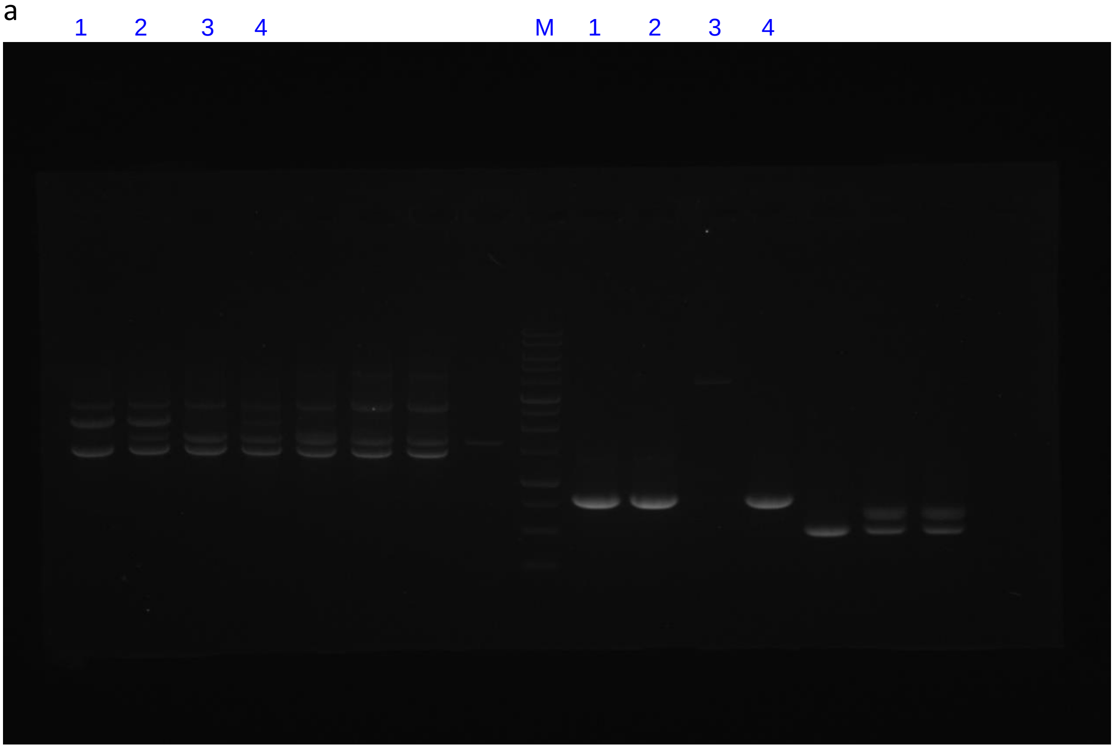

b

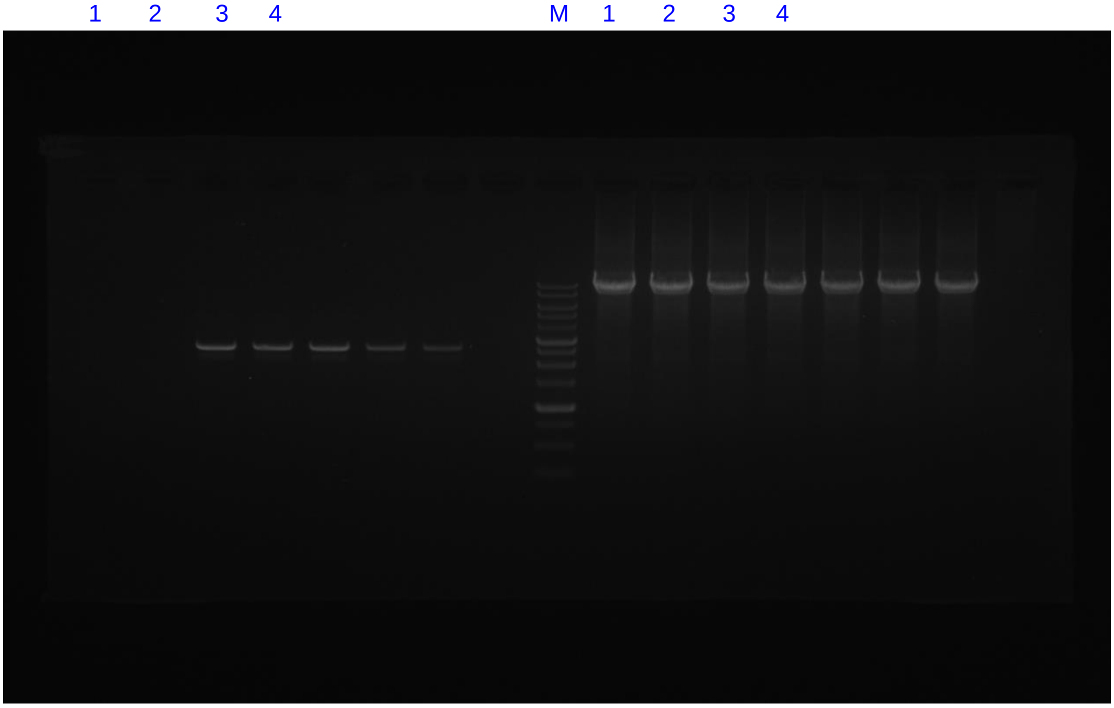

c

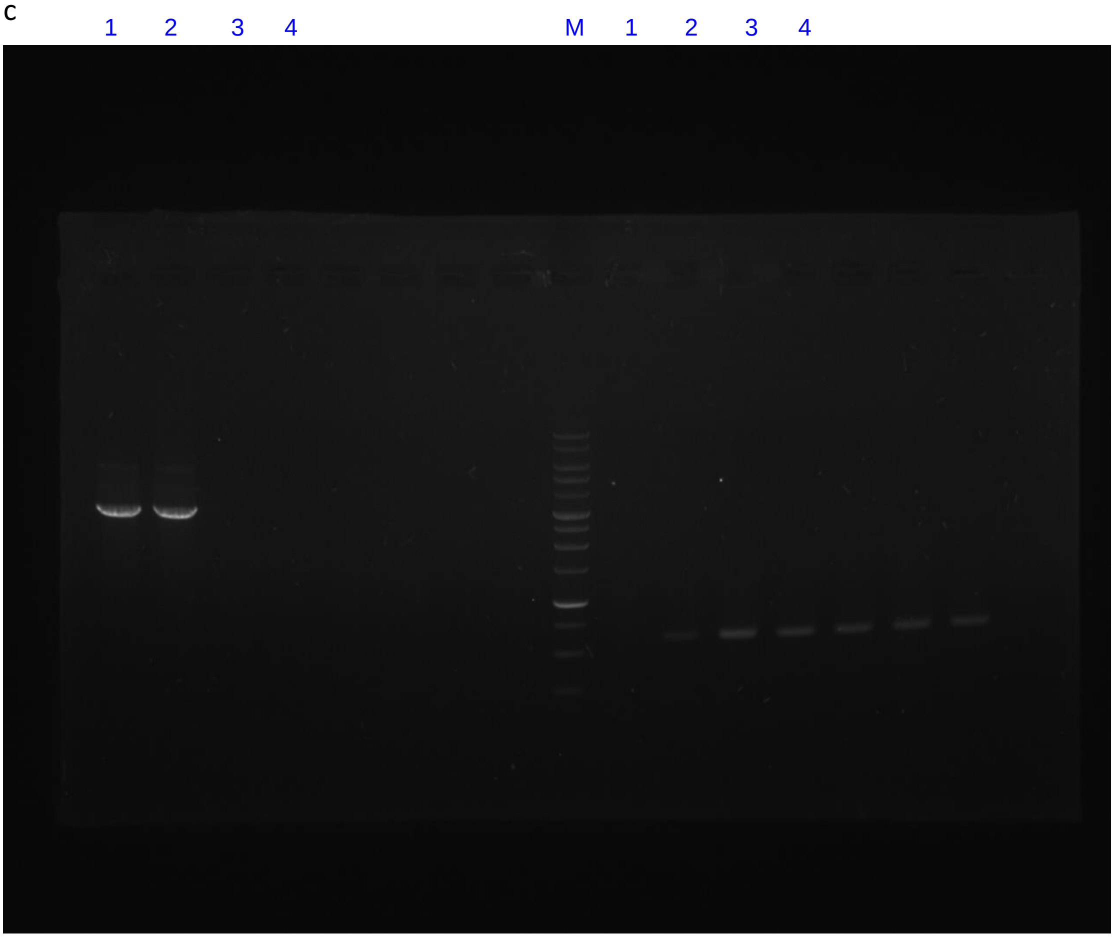

d

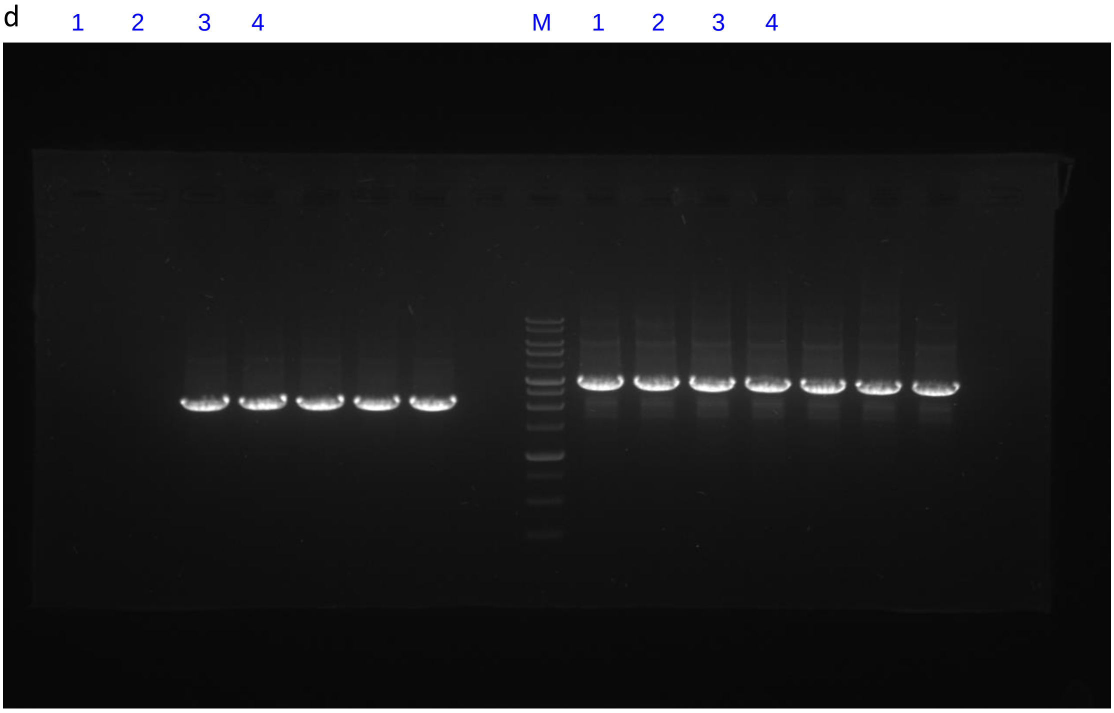

e 1 2 3 4 M 1 2 3 4

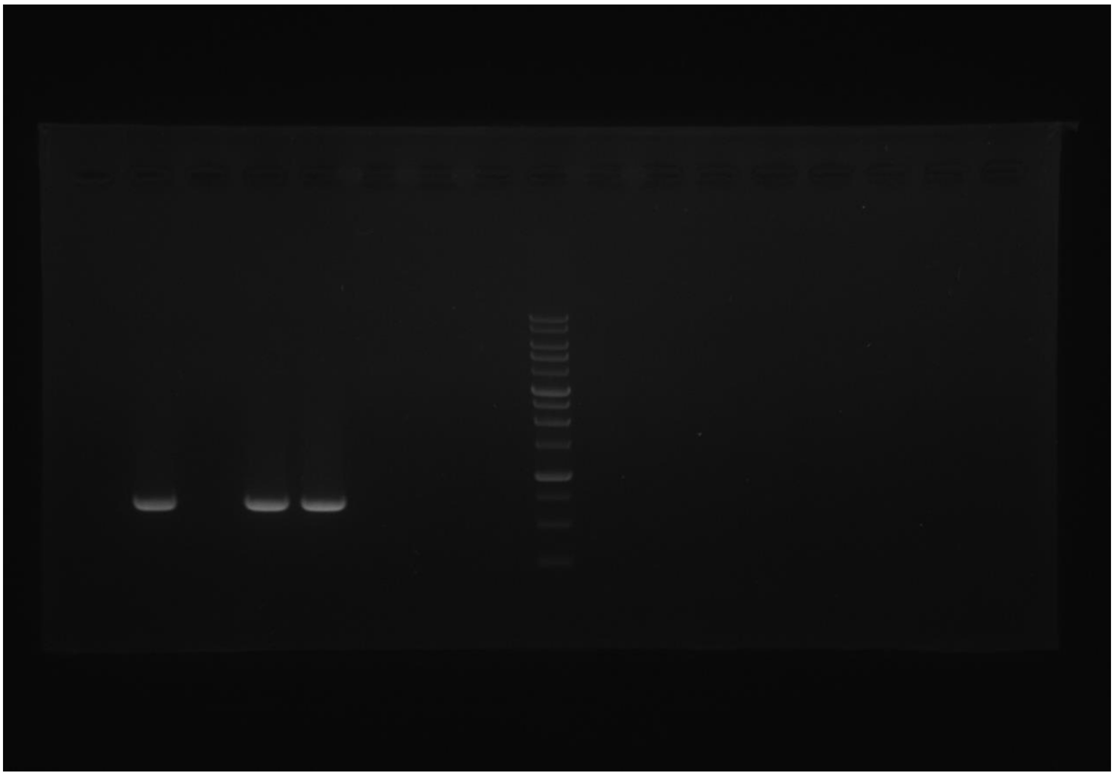

f (left) 1 2 3 4 M

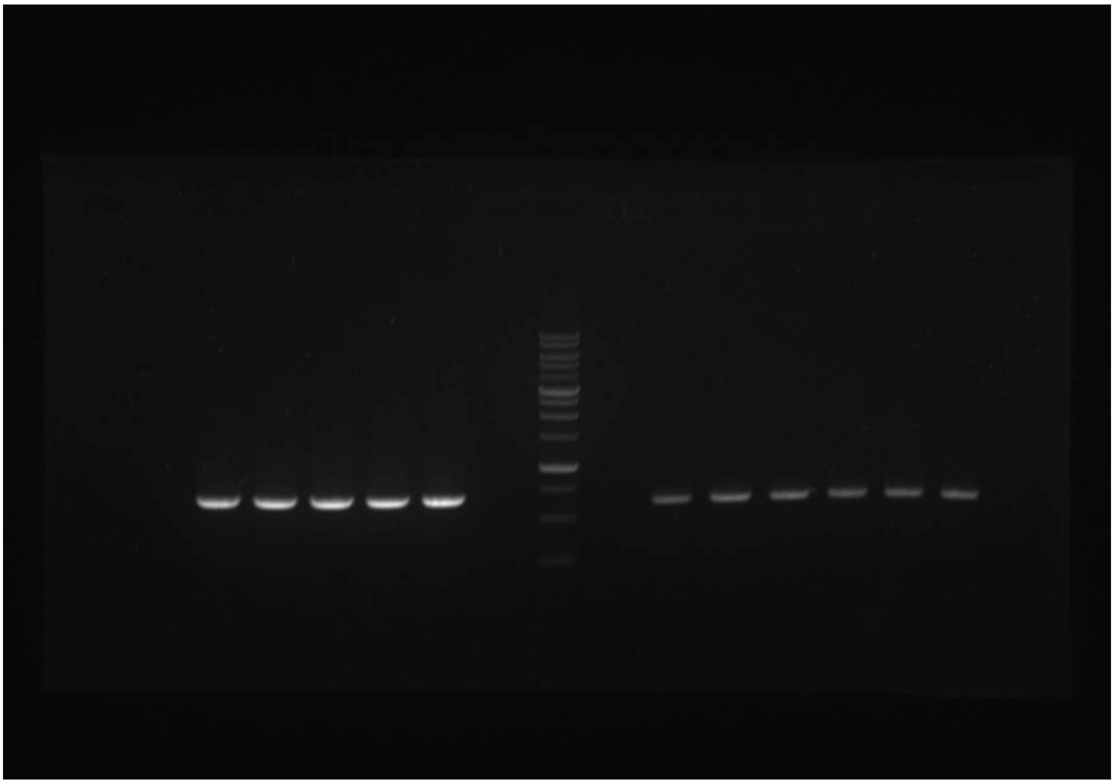

S6 Fig. Validation of the knockouts and knockins of donor DNAs to the target gene in

antibiotic-free strains by PCR. N: Negative control, M: DNA marker, Lane 1: αO4-I3ΔC, Lane 2: αO4-I4ΔC, Lane 3: αO4-I3ΔR, Lane 4: αO4-I4ΔR. (a) All gene cassettes were inserted to the chromosome and the genes inserted were validated by PCR, using the S1274F and S1276R primer pairs. The white font indicates the different fragment sizes of the transformed genes on the left side of the figure. We used the S1274F and MdsI-R2 primer pairs to confirm the three strains that were supposed to carry by the *MdsI* gene (right side of the figure). (b) The left side of the figure confirmed that the *GnTI* gene was inserted into the *URA3* gene position; it was checked by PCR using the URA3-F and GnTI-R primer pairs. The right side of the figure confirmed that the mating-type was retained on the α haploid. (c) The left side of the figure confirmed that the *MdsI* gene was inserted into the *URA3* gene; it was checked by PCR using the URA3-F and MdsI-R2 primer pairs. The right side of the figure confirmed that the *GnTI* gene was retained on the transformants by PCR using the S1274F and GnTI-R primer pairs. (d) Validation of the *Cas9* gene in the cell by PCR using the primer pair: S1274F and Cas9-M2R (left side of the figure). The white font indicates that *GnTII* was inserted into the *KU70* gene (right side of the figure). (e) Validation of the retention of *G418* in the transformants by PCR using the primer pair: SAD-F1 and G418-R (left side of the figure). Because the PCK protocol was used to knock out the *hygromycin* gene in all strains, no band of *hygromycin* was found in the chromosome by PCR using the primer pair: SAD-

F1 and Hyg-R. (f) The *zeocin* gene is adjacent to the *Cas9* gene and it was identified in those transformants carrying the *Cas9* gene.
